# Urban Environments and Obesity in Southeast Asia: A Systematic Review, Meta-Analysis and Meta-Regression

**DOI:** 10.1371/journal.pone.0113547

**Published:** 2014-11-26

**Authors:** Chaisiri Angkurawaranon, Wichuda Jiraporncharoen, Boriboon Chenthanakij, Pat Doyle, Dorothea Nitsch

**Affiliations:** 1 Department of Non-communicable Disease Epidemiology, Faculty of Epidemiology and Population Health, London School of Hygiene and Tropical Medicine, London, United Kingdom; 2 Department of Family Medicine, Faculty of Medicine, Chiang Mai University, Chiang Mai, Thailand; 3 Department of Emergency Medicine, Faculty of Medicine, Chiang Mai University, Chiang Mai, Thailand; University of Modena & Reggio Emilia, Italy

## Abstract

Many environmental factors contribute to the rise in prevalence of obesity in populations but one key driver is urbanization. Countries in Southeast (SE) Asia have undergone rapid changes in urbanization in recent decades. The aim of this study is to provide a systematic review of studies exploring the relationship between living in an urban or rural environment (urbanicity) and obesity in Southeast Asia. In particular, the review will investigate whether the associations are uniform across countries and ages, and by sex. The literature search was conducted up to June 2014 using five databases: EMBASE, PubMed, GlobalHealth, DigitalJournal and Open Grey. Forty-five articles representing eight of the eleven countries in SE Asia were included in the review. The review found a consistent positive association between urbanicity and obesity in countries of Southeast Asia, in all age groups and both genders. Regional differences between the associations are partly explained by gross national income (GNI). In countries with lower GNI per capita, the association between urbanicity and obesity was greater. Such findings have implications for policy makers. They imply that population level interventions need to be country or region specific, tailored to suit the current stage of economic development. In addition, less developed countries might be more vulnerable to the negative health impact of urbanization than more developed countries.

## Introduction

The increasing prevalence of obesity is a phenomenon happening worldwide, with global prevalence almost doubling since 1980 [1]. Previously considered an epidemic of developed countries, in recent years the growing burden of obesity has affected most regions, including Southeast Asia [2]. In Southeast Asia, like other parts of the world, obesity is considered one of the key risk factors for chronic and non-communicable disease [3],[4]. Its burden on health is reflected by the Global Burden of Disease project report [5]. In 1990, high BMI was ranked the 23^rd^ most important risk factor for SE Asia, and by 2010 it was 9^th^ [6].

Many environmental factors contribute to the rise in prevalence of obesity, but one key driver is urbanization [7]. The National Institute of Health defines urbanization as “the process whereby a society changes from a rural to an urban way of life. It refers also to the gradual increase in the proportion of people living in urban areas” [8].

The framework proposed by the International Obesity Taskforce has outlined possible causal pathways between urbanization and obesity [9]. In short, factors operating at the national and international level, such as urbanization, will influence the environment of the individual at the community and family level. Such environmental influences are likely to result in lower levels of physical activity and energy expenditure, coupled with a high energy and high fat diet [10].

Countries in Southeast (SE) Asia have undergone a rapid increase in urbanization in recent decades. The proportion living in an urban area rose from 15% to 32% between 1950 and 1990. By 2010, about 50% of the 600 million people in SE Asia were living in an urban area [11].

Since most studies on the impact of urbanization on health have focused on urban-rural differences [12], the aim of this study is to provide a systematic review of studies exploring the relationship between urban and rural environments (urbanicity) and obesity in Southeast Asia. In particular, the review will investigate whether the associations are uniform across countries and ages, and by sex.

## Methods

### Search strategies and procedures

The literature search was conducted up to June 2014 using five databases. Three standard international databases in the field of medicine, epidemiology and public health were used: EMBASE (from 1974), PubMed (from 1946), GlobalHealth (from 1910). We used one regional database: DigitalJournal (from 2007), which is an electronic journal database from SE Asian member countries and currently health science journals from Indonesia, Myanmar and Thailand can be searched electronically [13]. We used one database for grey literature and unpublished research: Open Grey (from 1980) [14]. Full articles of relevant abstracts were retrieved through the London School of Hygiene and Tropical Medicine and Chiang Mai University's network. We also conducted an additional cited-reference search from articles included in the review to pick up relevant published and unpublished articles. The search strategy using EMBASE can be found in the supporting document. (Table S1 in File S1)

### Inclusion and exclusion criteria

Criteria for articles to be included in the review were that they must:

- Have a clearly defined measure for an urban environment
- Have a defined measure of obesity
- Have a direct control group or comparison group such as a semi-urban or rural comparison group
- Report (or have data to be able to calculate) quantitative measures for the association between urban/non-urban environments and obesity
- Be published in English.

The eleven countries in SE Asia included in the review were Brunei Darussalam, Cambodia, Indonesia, Laos PDR, Malaysia, Myanmar, Philippines, Thailand, Timor-Leste, and Vietnam and Singapore. However, studies from Singapore were not expected, as the entire country was considered urban. As long as the inclusion criteria were met, we did not have restrictions on the type of study design included. We excluded any studies conducted outside the SE Asian region or studies with historical controls where the prevalence of obesity was measured at different time points within the same study.

### Screening and data extraction

Titles and abstracts were screened independently by two reviewers (CA and WJ) and classified into three subgroups:

- Clearly not relevant,
- Potentially relevant, and
- Relevant to review.

Studies that were classified as ‘clearly not relevant’ by both reviewers were excluded during the initial abstract screening process. Full text articles, which were classified as ‘potentially relevant’ or ‘relevant articles’ by one of the reviewers, were retrieved and reviewed by the lead author (CA). Reasons for exclusion (if relevant) were documented (Table S2 in File S1). Authors were contacted if full text articles were not retrievable or if additional information was needed to make a decision on inclusion or exclusion.

A small sample of literature included in the review was used to derive a standard data abstraction form. Information was collected on the lead author's name and year of publication, country and year of fieldwork, study design and sample size, characteristics of the study population (such as age and gender distribution), the definition of urban and non-urban/rural environment, and how the outcome of interest was defined and measured. In addition, the per capita Gross National Income (GNI) corresponding to the country and year of fieldwork was included. If year of fieldwork was not stated, it was assumed to be three years prior to year of publication. For the results section, prevalence and odds ratios were considered to be the main summary measures of interest. Information was also collected on which factors were controlled for if adjusted ratio measures of effect were reported. (Tables S3–S15 in File S1)

### Definition of variables for meta-regression

For each observation included in the meta-regression, the following definitions were used to define six variables:

1. Country of conduct: Based on the total number of observations from each country, the variable “country of conduct” was grouped according to geographical proximity and level of per capita GNI into four groups. They consisted of i) Malaysia and Philippines, ii) Thailand, iii) Vietnam and Laos, and iv) Indonesia and Timor-Leste
2. Per capita GNI (US dollar) corresponding to year of field work and county of conduct, as reported by the United Nations was obtained [15]. This was categorized into three groups: i) <1,500 dollars, ii) 1,500–2500 dollars iii) >2,500 dollars
3. Year of fieldwork was categorized into two groups, whether the study was conducted within i) ten years (2004–2013) or ii) earlier (up to 2003)
4. Age of study population was categorized into two groups: i) children (<18 years old) or ii) adults (≥18 years old)
5. Sex of study population was categorized into three groups: i) men only, ii) women only, or iii) both (results adjusted for sex)
6. Obesity classification: The obesity definition differed between individual studies. To explore the different obesity classifications as a source of heterogeneity, the variable “obesity classification” was categorized into three groups according to whether the study used a i) non BMI classification (using waist circumference), ii) a BMI classification (or corresponding percentiles) defining obesity as ≥23 kg/m^2^ or ≥25 kg/m^2^, or iii) a BMI classification (or corresponding percentiles) defining obesity as ≥30 kg/m^2^.

### Quality appraisal

The risk of bias within individual studies was assessed according to the Grading of Recommendations Assessment, Development, and Evaluation (GRADE) approach [16] as recommended by the Cochrane handbook [17]. In summary, information was collected on potential risk of i) selection bias, ii) confounding and residual confounding, and iii) information bias in the classification of an urban environment status and in the measurement of obesity. Information bias in exposure and outcome variables was also further assessed as likely to be differential or non-differential. Additional limitations of each study were recorded. We used the Preferred Reporting Items for Systematic Review and Meta-Analysis: The PRISMA statement as guidelines for reporting our results [18]. (Table S16 in File S1)

### Data analysis

For the results (odds ratios) of an individual article to be included in the meta-analysis, it must have been adjusted for age and sex, or stratified by sex and adjusted for age. If an article presented additional results adjusting for other covariates (such as socioeconomic status), we used the age and sex adjusted results. Additional adjustments could be considered over-adjustments for factors on the causal pathway between urbanicity and obesity.

We took the effect size (odds ratio) as reported by each article. If an article reported summary measures for more than one independent dataset, all available summary measures were used. If there was more than one summary measure reported from a single dataset, such as reporting by different gradients of urbanicity or with additional stratification by sex, we used the most reliable estimate (largest sample size) and the most conservative definition of obesity using BMI classifications. If odds ratios were not directly reported, when possible, we calculated crude odds ratios and CIs based on the proportions provided. However, crude odds ratios were not included in the meta-analysis, as these were not adjusted for age and sex.

High degrees of heterogeneity among studies were expected due to differences in the age distribution and regions of the study populations. Three main subgroup meta-analyses were pre-specified: i) analysis in children; ii) analysis in adult populations; and iii) analysis by country or countries.

In the absence of statistical heterogeneity, the fixed effect model using the inverse variance method was use to summarize the measures of effect. If there was evidence for heterogeneity, the DerSimonion and Laird approach for random effect models was used [19]. Heterogeneity was evaluated using Cochran's Q and I^2^ statistics. Combining results with high heterogeneity may lead to misleading results [20]. If there was high heterogeneity, I^2^>80%, the summary measures were displayed using Forest plots without combining effects. Funnel plots were used to evaluate publication bias for the meta-analyses.

### Sensitivity Analyses

Random effect meta-regression [21] was used to explore the role of age, gender, time periods, obesity classification, country of conduct, and stage of economic development as measured by per capita GNI as sources of heterogeneity for the association between urban/rural environment and obesity. In presence of potential publication bias, the trim and fill technique was used to explore the its impact [22]. Stata 12 was used in all analyses.

## Results

### Characteristics of studies

**Figure 1:**
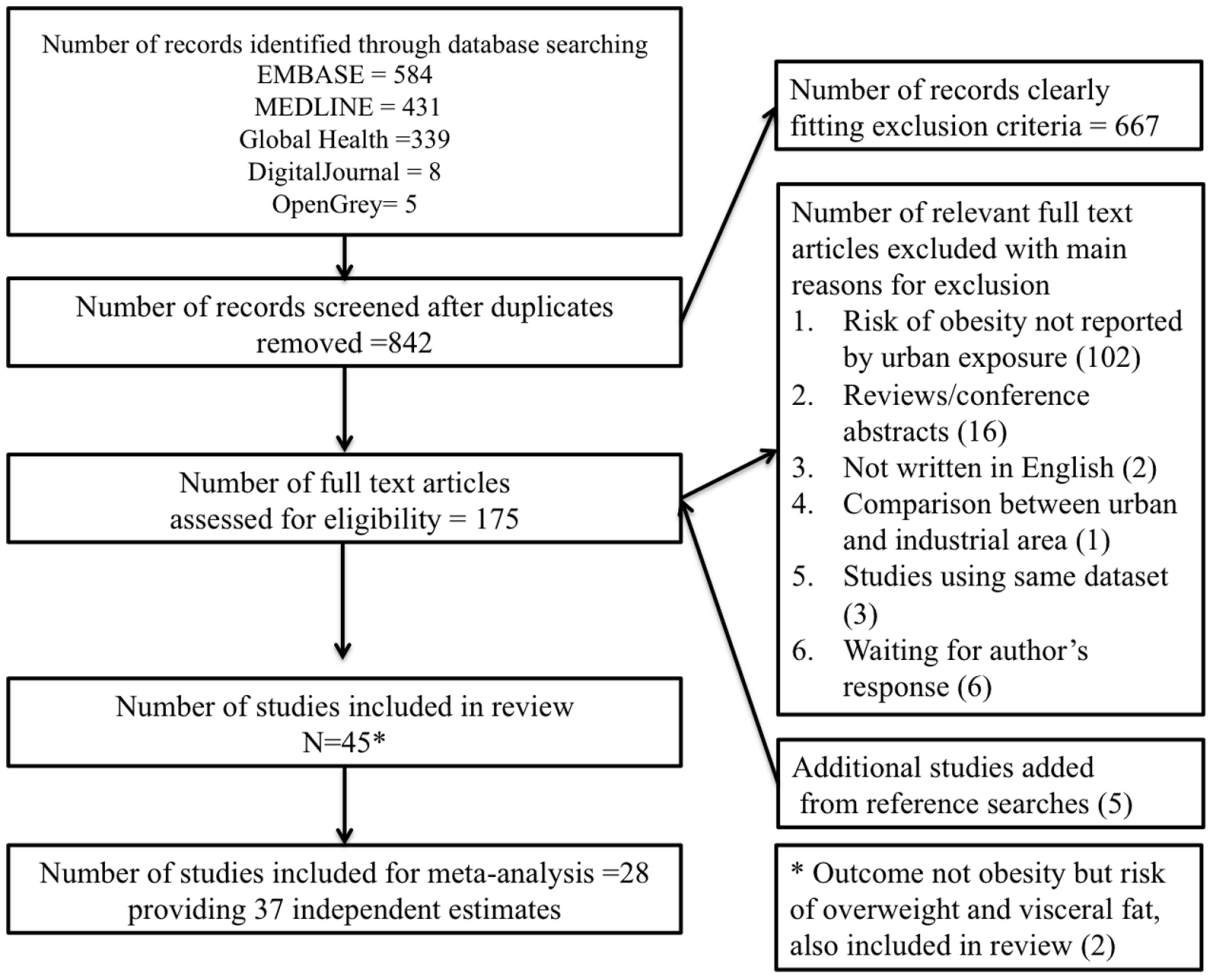
Flow chart of articles included in the review.

Forty-five studies met the inclusion criteria, and all were cross sectional in design (Figure 1). Eight of the eleven countries in SE Asia were covered by these 45 studies. Thirteen studies were from Malaysia, twelve from Vietnam, nine from Thailand, six from Indonesia, two from Laos, and one each from Philippines, Myanmar and Timor-Leste. Countries for which we found no studies were Brunei Darussalam, Cambodia and Singapore. Twenty-seven studies focused only on adults, seventeen focused only on children and/or adolescents (age <18 years old), and one study included both children and adults but reported estimates separately [23]. Two studies were published in1988 and 1992, the rest were published after 2000. Detailed characteristics of each study can be found in Tables S3–S8 in File S1.

### The urban environment and obesity in children

Eighteen studies included children, whose ages ranged between 2 and 18. Of these studies, six were from Vietnam [23],[24],[25],[26],[27],[28], six from Malaysia [29],[30],[31],[32],[33],[34], three from Thailand [35],[36],[37], two from Indonesia [38],[39] and one from Laos [40]. All classifications of obesity were age-and-gender specific, but studies differed in the criteria and the cutoff points used for obesity. Six studies used the International Obesity Task Force definition [24],[27],[29],[30],[38],[40], eleven studies used the World Health Organization's standard [23],[24],[25],[28],[31],[32],[33],[34],[35],[37],[39], and one study from Thailand used its own National standard [36].

Sixteen studies, consisting of at least one from each of the five countries presented, reported a significant association between an urban environment and obesity in children [23],[24],[25],[26],[27],[28],[31],[32],[33],[34],[35],[36],[37],[38],[39],[40]. The two studies that did not find a significant association were from Malaysia [29],[30]. Two studies explored a gradient effect between urbanicity and obesity. The study by Julia et al, conducted in Indonesia compared children in three different exposure groups: i) urban, ii) urban poor and iii) rural. The study found that although there were differences in obesity between urban and rural children, these differences were less pronounced when urban poor children were compared with rural children [38]. A gradient effect was also seen in the study by Tang et al, conducted in Vietnam [27]. The adjusted odds ratio for the wealthy urban population compared to the semi-rural and rural population was 5.53 (95% CI 2.42 to 14.16), and the odds ratio for less wealthy urban versus the semi-rural and rural population was 3.82 (95% CI 1.73 to 9.56). Individual results for each of the eighteen studies in children can be found in Tables S9–S11 in File S1.

**Figure 2:**
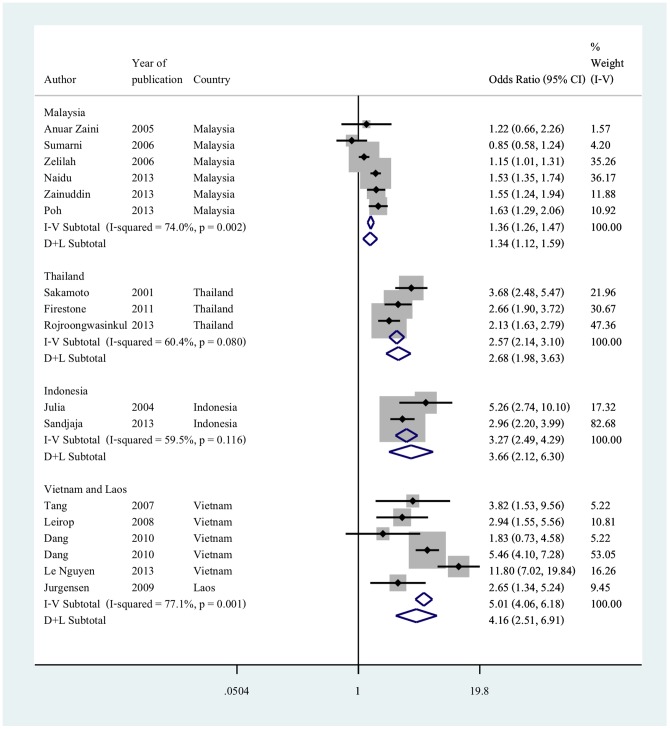
Adjusted odds ratio for living in an urban environment and obesity in children by country or countries. Reference group is living in a rural environment; Odds ratios are adjusted for age and sex; countries are grouped according to geographical proximities and gross national income per capita.

Sixteen of the eighteen studies were included in the meta-analysis [24],[25] [27] [28] [29],[30],[31],[32],[33],[34],[35],[36],[37],[38],[39],[40]. The random effect estimates gave a pooled odds ratio of 1.34 (95% CI 1.12 to 1.59) in studies from Malaysia and 2.68 (95% CI 1.98 to 3.63) in studies from Thailand. The pooled odds ratio was 3.66 (95% CI 2.12 to 6.30) in studies from Indonesia and 4.16 (95% CI 2.51 to 6.91) in studies from Vietnam and Laos (Figure 2).

### The urban environment and obesity in adults

Twenty-eight studies included adults, whose ages ranged between 18 to over 80. Of these studies, seven each were from Vietnam [23],[41],[42],[43],[44],[45],[46] and Malaysia [47],[48],[49],[50],[51],[52],[53], six were from Thailand [54],[55],[56],[57],[58],[59], four from Indonesia [60],[61],[62],[63] and one each from the Philippines [64], Timor-Leste [65] and Myanmar [66] and Laos [67]. Twelve studies, representing Vietnam [23],[41],[44], Thailand [54],[55],[56],[57],[59], Malaysia [47],[49],[50] and Timor-Leste [65], were considered nationally representative of the adult population of these nations. Other study populations which were not considered representative of the national populations included an indigenous population in Malaysia [48], Thai university students [58] and an elderly Malaysian and Laotian populations [51],[67].

Most studies reported obesity as measured by BMI, although using different cut-off points to define obesity. Two reported waist circumference as the only measure of obesity [49],[54]. Fuke et al studied visceral fat in adults from Indonesia with normal BMI and did not find an association between urban-rural differences and visceral fat [62]. Seven studies did not find an association between an urban environment and obesity in adults, four from Malaysia [47],[49],[52],[53] and one each from Vietnam [43], Indonesia [63] and Philippines [64]. Of these seven studies, only two studies were adjusted for both age and gender [53],[64]. Rasiah et al additionally adjusted for level of education [53]. Dahly et al used an urbanicity score as their exposure rather than directly comparing outcomes by urban and rural status [64]. Four studies looked for a gradient effect between urbanicty and obesity in adults [42],[43],[60],[64], all of which reported higher prevalence of obesity in populations with greater levels of urbanization. Results for each of the twenty-eight studies in adults can be found in Tables S12–S15 in File S1.

**Figure 3:**
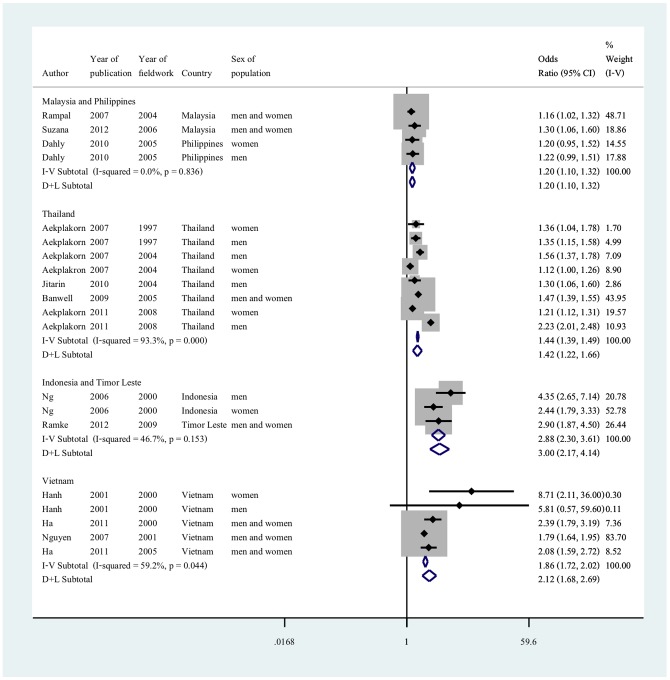
Adjusted odds ratio for living in an urban environment and obesity in adults by country or countries. Reference group is living in a rural environment; Odds ratios are adjusted for age and sex (or adjusted for age if stratified by sex); countries are grouped according to geographical proximities and gross national income per capita.

Twelve studies, from six nations, met the criteria for meta-analysis by reporting age and sex adjusted odds ratio. The six nations represented were grouped into four groups taking into consideration geographical proximities and/or similar gross national income level: i) Malaysia and Philippines, ii) Thailand, iii) Indonesia and Timor-Leste and iv) Vietnam (Figure 3). In studies from Malaysia and Philippines, there was no heterogeneity between the results (I^2^ = 0, p = 0.836). The pooled random effect estimates gave an odds ratio of 1.20 (95% CI 1.10 to 1.32). All adjusted estimates between urbanicity and obesity from Thailand were statistically significant, but had very high heterogeneity (I^2^ = 93.3, p<0.001). The results from Indonesia and Timor-Leste showed moderate heterogeneity (I^2^ 46.7, p = 0.153), the random effect model gave an adjusted odds ratio of 3.0 (95% CI 2.17 to 4.14). There was moderate heterogeneity between the results from Vietnam (I^2^ = 59.2, p = 0.044), and the pooled random effect odds ratio was 2.12 (95% CI 1.68 to 2.69).

### Sources of heterogeneity: Results from Meta-regression

**Figure 4:**
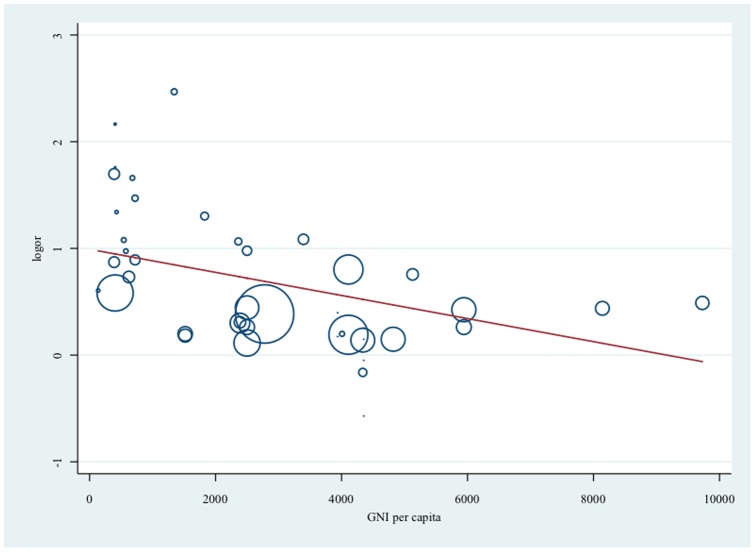
Association (log odds ratio) between living in an urban environment and obesity by GNI per capita. Size of circles reflects sample size. Higher log odds ratio (logor) reflect larger effect size for living in an urban environment and obesity; gross national income (GNI) per capita in US dollar corresponding to year and country of fieldwork; Reference group is living in a rural environment.

Twenty-eight studies, contributing thirty-seven independent age and sex-adjusted estimates, were included for meta-regression. Exploring six potential sources of heterogeneity separately, results suggested that there was heterogeneity in the association between urbanicity and obesity both within country and between countries of SE Asia (Table 1). Country setting drove much of the heterogeneity in these estimates, which in turn may be related to the economic output of that country at the time the studies were conducted. The pooled measure of association between urbanicity and obesity in countries such as Malaysia and Philippines (OR 1.29, 95% CI 1.14 to 1.45) was smaller than the association seen in lower income countries such as Indonesia and Timor-Leste (OR 3.14, 95% CI 2.22 to 4.46) (Table 1). Figure 4 presents the association between urbanicity and obesity by GNI per capita. There was strong evidence that the association is greater when GNI per capita was smaller. No other sources of heterogeneity were statistically significant but there was some weak evidence that effect size in children may be larger than adults (p = 0.07) (Table 1). When including per capita GNI, country/countries of conduct, and other possible sources of heterogeneity (age and sex of study population, whether the study was conducted within the past ten years or before, and the type of BMI classification for obesity used), these six variables together were able to explain 22.4% of the heterogeneity between results.

**Table 1:** Adjusted odds ratios (OR) for living in an urban environment and obesity using stratification by country/countries, per capita GNI, year of fieldwork, sex, age of study population and criteria for obesity.

Stratification	Number of observations	OR for living in an urban environment (95% CI)	P-value	I^2^	p-values*	F-ratio (p-value)**
**None**	37	1.99 (1.64 to 2.41)	<0.001	92.1%	<0.001	–
**Country/countries**						12.16 (<0.001)
Philippines and Malaysia	10	1.29 (1.14 to 1.45)	0.001	62.8%	<0.001	
Thailand	11	1.66 (1.30 to 2.11)	0.001	93.2%	<0.001	
Vietnam and Laos	11	3.36 (2.14 to 5.27)	<0.001	90.6%	<0.001	
Indonesia and Timor-Leste	5	3.14 (2.22 to 4.46)	0.001	40.4%	<0.001	
**Per capita GNI (US dollars)**						12.00 (<0.001)
<1,500	14	3.42 (2.42 to 4.84)	<0.001	89.4%	<0.001	
1,500–2,500	10	1.62 (1.20 to 2.18)	<0.001	86.7%	<0.001	
>2,500	13	1.50 (1.23 to 1.82)	0.01	91.9%	<0.001	
**Year of field work**						0.78 (0.383)
2004 to 2013	20	1.85 (1.45 to 2.37)	<0.001	92.4%	<0.001	
Up to 2003	17	2.22 (1.60 to 3.09)	<0.001	91.9%	<0.001	
**Sex of study population**						0.94 (0.407)
Men only	7	1.76 (1.14 to 2.73)	0.020	90.8%	<0.001	
Women only	6	1.47 (0.89 to 2.43)	0.106	82.8%	<0.001	
Both	24	2.19 (1.70 to 2.81)	<0.001	92.2%	<0.001	
**Age of population**						3.57 (0.067)
Children	17	2.43 (1.72 to 3.43)	<0.001	92.9%	<0.001	
Adults	20	1.65 (1.36 to 1.99)	<0.001	90.9%	<0.001	
**Obesity classifcation**						1.18 (0.318)
Non BMI classifciation (using WC)	3	2.10 (0.53 to 8.28)	0.145	98.0%	<0.001	
Obesity defined BMI≥23 or 25	29	2.13 (1.69 to 2.67)	<0.001	91.1%	<0.001	
Obesity defined as BMI≥30	5	1.39 (0.90 to 2.16)	0.104	80.9%	<.0.001	

Twenty eight studies contributed to 37 independent age and sex adjusted estimates (Figure 1); Reference group is living in a rural environment; GNI gross national income; WC waist circumference;

* p-value for heterogeneity chi-square;

** Likelihood ratio test for heterogeneity between subgroup by meta-regression, providing F-ratio and p-values.

### Sensitivity analysis

The funnel plots suggested that there was potential for publication bias (Figure S1 and Figure S2). However, sensitivity analysis using the trim-and fill technique did not materially alter any of the results seen. (Table S17 in File S1)

## Discussion

To our knowledge, this is the first systematic review to examine the association between living in an urban environment and obesity in SE Asia. The review found consistent positive associations between urbanicity and obesity in countries of Southeast Asia, in both genders and all age groups. We found that different country settings contributed strongly to the source of heterogeneity between the estimates. There was strong evidence that the association between urban environments and obesity is modified by the country's GNI per capita and this partly explained the observed heterogeneity of the estimates.

### Sources of Heterogeneity: Regional differences

Associations between urban environments and obesity were expected to vary between countries because of different cultures, and varying political and socioeconomic environments. When the data were grouped according to country or countries with close geographic proximity and similar economic status, some of the observed heterogeneity decreased. The notable exception was Thailand. However, these studies differed in other ways: one was conducted in university students [58], one used abdominal obesity [56] and another used a cut of point of BMI≥23 kg/m^2^ [33] as the outcome.

A systematic review from developed countries exploring the role of geographic environment on cardiometabolic risk factors, such as obesity, was conducted by Leal and Chaix [68]. The review found that living in a rural environment and areas with lower socioeconomic level was associated with higher BMI but did not look at the effect modification between these two exposures. The review by Leal and Chaix may not be generalizable to developing countries of SE Asia which may explain why we found the opposite, i.e. that the association between living in an urban environment and obesity was positive. Monteiro et al combined nationally representative data on women from 37 developing countries to examine the association between obesity and inequality [69]. The study found that there was interaction between the women's socioeconomic status (SES) and the country's Gross Nation Product (GNP), which was seen as a measure of the environmental level of economic development. Specifically, if the country's GNP per capita was less than 2,500 dollars, high SES was positively associated with obesity. If the country's GNP was greater than 2,500 dollars, the risk of obesity was highest for the poor. These observations support the findings of our review.

One explanation for an interaction between income (or SES) and urbanization (as a development process) on obesity could be sociocultural and behavioral in nature. It could be that in less developed countries people with higher incomes have easier access to a plentiful food supply. Whereas in more developed countries, people with higher income have options to counter-balance the impact of an obesogenic environment [70]. The ‘developmental origins’ theory [71] can also be used to help explain such interactions. If early life under-nutrition is associated with rapid weight gain in childhood and risk of obesity in adults, less developed countries would be more vulnerable to the obesogenic impact of urbanization.

### Other sources of heterogeneity between studies

This review also examined whether the association between urban environment and obesity differed between children and adults, and by gender. We found some very weak evidence that the effects were more pronounced in children than in adults. Literature has suggested that for childhood obesity, growth and puberty may interact with the obesogenic environment associated with urbanization [72]. The size of the effect may be reduced for children around puberty as they experience a growth spurt. In SE Asia where the prevalence of obesity is relatively low, there could be a cultural expectation for women to remain slim [2]. However, we did not find evidence that gender modified the association between urban environment and obesity. The current meta-analysis may be underpowered to detect an interaction with gender, and the high heterogeneity between studies could limit generalization of a potential finding.

### Strengths and limitations

The review had several limitations. It is possible that not all relevant articles on urban environment and obesity in SE Asia were included in the review. Omitted studies could have been published in other formats such as country reports or could have been published in other databases or in other languages. All studies were of cross-sectional design which, in principle, is susceptible to reverse causality. However, it is difficult to imagine how obesity would drive urbanization. All studies, except one [64], included in this review examined the association between an urban environment and obesity through comparing outcomes in rural and urban settings. Such comparisons do not reflect urbanization as a process, and offer little insight into the underlying mechanisms for the associations found. The failure to account for length of stay in an urban area, transient migration (urban migration to work during parts of the year) and economic diversity within urban areas may have caused bias in the estimates and limit the interpretation of findings. However, even if these biases existed, they are likely to lead to an underestimate of the association between exposure to an urban environment and obesity.

The strengths of the study include conducting the literature search using a regional SE Asian database and exploring the sources of heterogeneity using meta-regression. There was good inter-rater agreement between the reviewers (Kappa 0.85) (Table S18 in File S1). We also reviewed all articles classified as ‘potentially relevant’ or ‘relevant’ irrespective of agreement between the reviewers. Although there was potential for publication bias, our results did not materially alter in the sensitivity analysis. The evidence for interactions between urban living and obesity with the country's GNI per capita was unlikely to be spurious effects due to poorly conducted studies as most studies included in the meta-analysis were assessed to be at low risk of bias (Table S19 and Table S20 in File S1).

### Unanswered questions and future research

A better quantification of specific environmental characteristics, carrying out migrant studies, and taking a life-course approach to examine the development of obesity within individuals over time would be useful to enable understanding of the mechanisms underlying the link between urban environments and obesity in this region [72],[73].

## Conclusions and Policy Implications

This systematic review found a consistent positive association between living in an urban environment and obesity in countries of Southeast Asia, across all age groups and both genders. Regional differences between the associations are partly explained by gross national income (GNI). The association between urban environments and obesity was stronger in countries with lower GNI per capita. Exposure to an urban environment was associated with 29% higher odds of obesity in Malaysia and Philippines (pooled OR 1.29, 95% CI 1.14 to 1.45). In countries with lower GNI such as Vietnam and Laos, exposure to urban environment was associated with a three-fold increase in obesity (pooled OR 3.36, 95% CI 2.14 to 5.27).

Our findings imply that population level interventions need to be country or region specific, tailored to suit the stage of economic development [74]. Developing countries such as those in SE Asia may be more vulnerable to the negative health impacts of urbanization than more developed countries. A recent report from Malaysia in 2013 highlighted that economic growth has accelerated the problem of obesity though availability of high calorie diets and decreased physical activity in the population. The authors suggested that the creation of healthy infrastructure for active transportation, protection of natural environment, along with healthy and affordable food resources are vital for sustainable economic development [75]. Environmental interventions are recognized as a promising strategy to combat obesity and other obesity-related conditions [76],[77]. School based interventions have been successful in reducing obesity in Singapore [78].Other countries in SE Asia, such as Thailand and Indonesia, have also made progress by adopting population approaches to prevent and control obesity [79].

## Supporting Information

Figure S1 **Funnel plots of results included in meta-analysis.**
(TIFF)

Figure S2 **Funnel plots of results included in meta-analysis by country/countries.**
(TIFF)

Data S1 **Data extraction worksheet.**
(XLSX)

File S1 **Supporting tables.** Table S1: Search strategy using EMBASE. Table S2: List of excluded articles by main reasons for exclusion. Table S3: Study characteristics of studies conducted in children (<18) from Malaysia, Thailand and Indonesia. Table S4: Study characteristics of studies conducted in children (<18) from Laos and Vietnam. Table S5: Study characteristics of studies conducted in adults from Malaysia and Philippines. Table S6: Study characteristics of studies conducted in adults from Thailand. Table S7: Study characteristics of studies conducted in adults from Indonesia and Timor-Leste. Table S8: Study characteristics of studies conducted in adults from Laos, Vietnam and Myanmar. Table S9: Results of studies conducted in children from Malaysia. Table S10: Results of studies conducted in children from Thailand and Indonesia. Table S11: Results of studies conducted in children from Laos and Vietnam. Table S12: Results of studies conducted in adults from Malaysia and Philippines. Table S13: Results of studies conducted in adults from Thailand. Table S14: Results of studies conducted in adults from Indonesia and Timor-Leste. Table S15: Results of studies conducted in adults from Laos, Vietnam and Myanmar. Table S16: PRISMA checklist. Table S17: Sensitivity analysis: Results from random effect meta-regression and trim and fill technique. Table S18: Inter-rater agreement from abstract screening. Table S19: Summary of potential biases within studies among children. Table S20: Summary of potential biases within studies among adults.
(DOCX)
