# Supplementary figures and images for: Urban Environments and Obesity in Southeast Asia: A Systematic Review, Meta-Analysis and Meta-Regression
Source: PLoS One. 2014 Nov 26;9(11):e113547. doi: 10.1371/journal.pone.0113547 (PMC4245122; doi:10.1371/journal.pone.0113547)

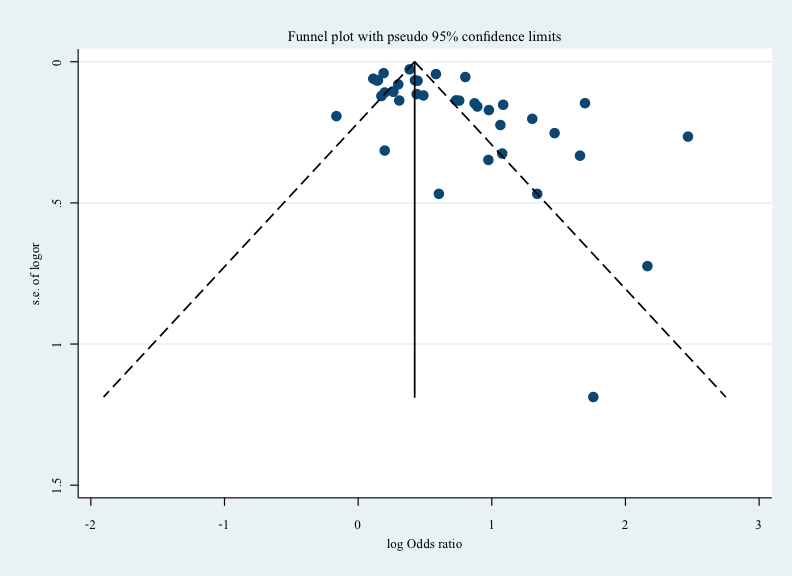

Supplement: Figure S1 — Funnel plots of results included in meta-analysis. (TIFF) [file pone.0113547.s001.tiff]

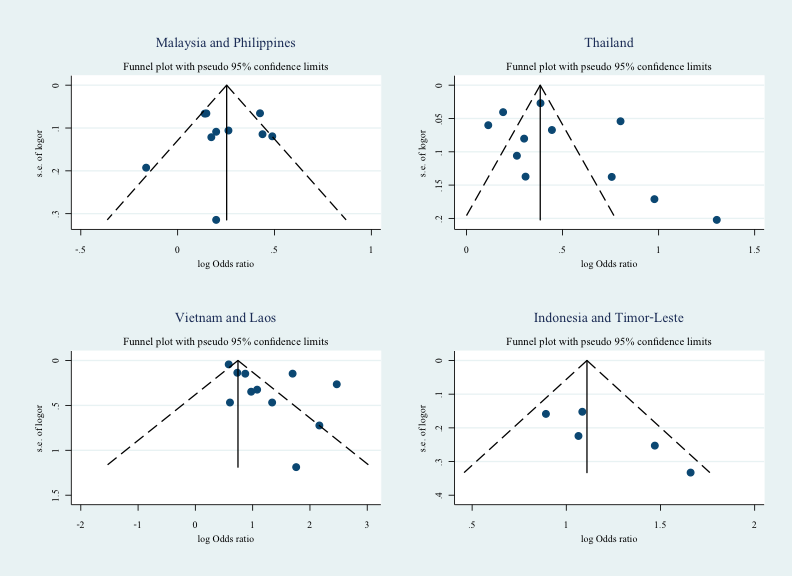

Supplement: Figure S2 — Funnel plots of results included in meta-analysis by country/countries. (TIFF) [file pone.0113547.s002.tiff]
