# Supplementary material for: Urban Environments and Obesity in Southeast Asia: A Systematic Review, Meta-Analysis and Meta-Regression
Source: PLoS One. 2014 Nov 26;9(11):e113547. doi: 10.1371/journal.pone.0113547 (PMC4245122; doi:10.1371/journal.pone.0113547)
Supplement: File S1 — Supporting tables. Table S1: Search strategy using EMBASE. Table S2: List of excluded articles by main reasons for exclusion. Table S3: Study characteristics of studies conducted in children (<18) from Malaysia, Thailand and Indonesia. Table S4: Study characteristics of studies conducted in children (<18) from Laos and Vietnam. Table S5: Study characteristics of studies conducted in adults from Malaysia and Philippines. Table S6: Study characteristics of studies conducted in adults from Thailand. Table S7: Study characteristics of studies conducted in adults from Indonesia and Timor-Leste. Table S8: Study characteristics of studies conducted in adults from Laos, Vietnam and Myanmar. Table S9: Results of studies conducted in children from Malaysia. Table S10: Results of studies conducted in children from Thailand and Indonesia. Table S11: Results of studies conducted in children from Laos and Vietnam. Table S12: Results of studies conducted in adults from Malaysia and Philippines. Table S13: Results of studies conducted in adults from Thailand. Table S14: Results of studies conducted in adults from Indonesia and Timor-Leste. Table S15: Results of studies conducted in adults from Laos, Vietnam and Myanmar. Table S16: PRISMA checklist. Table S17: Sensitivity analysis: Results from random effect meta-regression and trim and fill technique. Table S18: Inter-rater agreement from abstract screening. Table S19: Summary of potential biases within studies among children. Table S20: Summary of potential biases within studies among adults. (DOCX) [file pone.0113547.s004.docx]

# Table S1: Search strategy using EMBASE

|  | Searches |
| --- | --- |
| 1 | Southeast asia or SE Asia or SE-asia or South-east asia |
| 2 | brunei or myanmar or burma or cambodia or east timor or indonesia or laos or malaysia or philippines or singapore or thailand or vietnam |
| 3 | Southeast Asia/ OR Myanmar/ OR Cambodia/ OR Timor/ OR Indonesia/ OR Laos/ OR Malaysia/ OR Philippines/ OR Singapore/ OR Vietnam/ OR Thailand/ OR Brunei |
| 4 | 1 or 2 or 3 |
| 5 | Urbani#ation or urbanicity or urban or rural or rurality |
| 6 | Urban adj3 rural |
| 7 | ( urban adj3 migra*) OR (rural adj3 migra*) |
| 8 | migration? or migrant? |
| 9 | urbanization/ or urban population/ or urban rural difference/ or rural population/ |
| 10 | migration/ |
| 11 | Or/5-10 |
| 12 | Obesity or obese or overweight |
| 13 | BMI or body mass index or body-mass-index or waist circumference or (waist adj3 hip adj3 ratio) |
| 14 | exp abdominal obesity/ or exp obesity/ |
| 15 | exp body mass/ or exp body height/ or exp body weight/ |
| 16 | Or/12-15 |
| 17 | 16 and 11 and 4 |
| 18 | 17 and “human” [subjects] |

# Table S2: Lists of excluded articles by mains reasons for exclusion

1. **Risk of obesity not reported by urban exposure (N=102)**

Abdur R, Lutfun N, Hoang Van M, Ng N, Sanjay J, et al. (2009) Social factors and overweight: evidence from nine Asian INDEPTH Network sites. (Special Issue: Risk factors for chronic non-communicable disease: the burden in Asian INDEPTH health and demographic surveillance sites.). Global Health Action 2.

Adair L, Popkin BM, VanDerslice J, Akin J, Guilkey D, et al. (1993) Growth dynamics during the first two years of life: A prospective study in the Philippines. European Journal of Clinical Nutrition 47: 42-51.

Adair LS (2001) Size at birth predicts age at menarche. Pediatrics 107: E59.

Adair LS (2004) Dramatic rise in overweight and obesity in adult filipino women and risk of hypertension. Obesity research 12: 1335-1341.

Adair LS and Guilkey DK (1997) Age-specific determinants of stunting in Filipino children. Journal of Nutrition 127: 314-320.

Adair LS and Popkin BM (1992) Prolonged lactation contributes to depletion of maternal energy reserves in Filipino women. Journal of Nutrition 122: 1643-1655.

Adair LS and Popkin BM (1996) Low birth weight reduces the likelihood of breast-feeding among Filipino infants. Journal of Nutrition 126: 103-112.

Aekplakorn W, Abbott-Klafter J, Premgamone A, Dhanamun B, Chaikittiporn C, et al. (2007) Prevalence and management of diabetes and associated risk factors by regions of Thailand: Third National Health Examination Survey 2004. Diabetes Care 30: 2007-2012.

Ahmed SM, Abdullahel H, Abdur R, Ali A, Sanjay J, et al. (2009) Clustering of chronic non-communicable disease risk factors among selected Asian populations: levels and determinants. (Special Issue: Risk factors for chronic non-communicable disease: the burden in Asian INDEPTH health and demographic surveillance sites.). Global Health Action 2.

Ali O, Tan TT, Sakinah O, Khalid BA, Wu LL, et al. (1993) Prevalence of NIDDM and impaired glucose tolerance in aborigines and Malays in Malaysia and their relationship to sociodemographic, health, and nutritional factors. Diabetes Care 16: 68-75.

Anuar Zaini MZ, Lim CT, Low WY and Harun F (2005) Effects of nutritional status on academic performance of Malaysian primary school children. Asia-Pacific Journal of Public Health 17: 81-87.

Aramrattana A, Limpijarnkit L, Leelapat P, Sriphrapradang A, Mangklabruks A, et al. (2002) Difference in goiter rates between two areas in Mae Hong Son province despite an equally sufficient iodine supply. Journal of the Medical Association of Thailand 85: 831-838.

Baltazar JC, Ancheta CA, Aban IB, Fernando RE and Baquilod MM (2004) Prevalence and correlates of diabetes mellitus and impaired glucose tolerance among adults in Luzon, Philippines. Diabetes Research and Clinical Practice 64: 107-115.

Benefice E, Levi P and Banouvong P (2012) Progressive growth deterioration in a context of nutritional transition: A case study from Vientiane (Lao PDR). Annals of Human Biology 39: 239-246.

Boffetta P, McLerran D, Chen Y, Inoue M, Sinha R, et al. (2011) Body mass index and diabetes in Asia: a cross-sectional pooled analysis of 900,000 individuals in the Asia cohort consortium. PLoS ONE 51.

Branch KE, Adair LS and Agustin S (2010) Prevalence and predictors of body image discrepancy and dissatisfaction among Filipino young adults. FASEB Journal Conference: Experimental Biology 20100424.

Casson RJ, Abraham LM, Newland HS, Muecke J, Sullivan T, et al. (2008) Corneal thickness and intraocular pressure in a nonglaucomatous Burmese population: the Meiktila Eye Study. Archives of Ophthalmology 126: 981-985.

Collins AE, Pakiz B and Rock CL (2008) Factors associated with obesity in Indonesian adolescents. International Journal of Pediatric Obesity 3: 58-64.

Craven KL and Hawks SR (2006) Cultural and western influences on the nutrition transition in Thailand. Promotion & education 13: 14-20.

Dahly DL, Gordon-Larsen P, Emch M, Borja J and Adair LS (2013) The spatial distribution of overweight and obesity among a birth cohort of young adult Filipinos (Cebu Philippines, 2005): An application of the Kulldorff spatial scan statistic. Nutrition and Diabetes 3.

Fatmah Y (2011) Visceral fat with its risk factors amongst the Indonesian Javanese elderly. Journal of Public Health and Epidemiology 3: 155-161.

Fitzpatrick AL, Ngo QV, Ly KA, Ton TGN, Longstreth WT, et al. (2012) Symptoms and risk factors for stroke in a community-based observational sample in Viet Nam. Journal of Epidemiology and Global Health 2: 155-163.

Giay T and Khoi HH (1994) Use of body mass index in the assessment of adult nutritional status in Vietnam. European Journal of Clinical Nutrition 48: S124-S130.

Gopalakrishnan S, Ganeshkumar P, Prakash MV, Christopher and Amalraj V (2012) Prevalence of overweight/obesity among the medical students, Malaysia. Med J Malaysia 67: 442-444.

Ha Huy K and Tu G (1995) Use of food intake and body mass index (BMI) in the assessment of adult nutritional status in Viet Nam including a maternal-child analysis. Asia Pacific Journal of Clinical Nutrition 4: 220-224.

He J, Neal B, Gu D, Suriyawongpaisal P, Xin X, et al. (2004) International collaborative study of cardiovascular disease in Asia: design, rationale, and preliminary results. Ethnicity & disease 14: 260-268.

Heinrich-Weltzien R, Monse B, Benzian H, Heinrich J and Kromeyer-Hauschild K (2013) Association of dental caries and weight status in 6- to 7-year-old Filipino children. Clinical Oral Investigations 17: 1515-1523.

Henry CJK, Webster-Gandy J and Varakamin C (2001) A comparison of physical activity levels in two contrasting elderly populations in Thailand. American Journal of Human Biology 13: 310-315.

Hien VTT, Khan NC, Lam NT, Mal LB, Le DN, et al. (2005) Determining the prevalence of osteoporosis and related factors using quantitative ultrasound in Vietnamese adult women. American Journal of Epidemiology 161: 824-830.

Hirata M, Kuropakornpong V, Funahara Y, Kamae I and Sato S (1998) Obesity among school children in a province of southern Thailand and its association with socioeconomic status. Environmental Health and Preventive Medicine 3: 67-72.

Hong TK, Trang NH and Dibley MJ (2013) Changes in adiposity indicators of Ho Chi Minh City adolescents in a 5-year prospective cohort study. International Journal of Obesity 37: 1261-1267.

Ishine M, Wada T, Sakagami T, Pham Tien D, Tranc Duc V, et al. (2005) Comprehensive geriatric assessment for community-dwelling elderly in Asia compared with those in Japan: III. Phuto in Vietnam. Geriatrics and Gerontology International 5: 115-121.

Ismail MN, Chee SS, Nawawi H, Yusoff K, Lim TO, et al. (2002) Obesity in Malaysia. Obesity reviews : an official journal of the International Association for the Study of Obesity 3: 203-208.

Jaruratanasirikul S, Sangsupawanich P, Koranantakul O, Chanvitan P, Sriplung H, et al. (2009) Influence of maternal nutrient intake and weight gain on neonatal birth weight: A prospective cohort study in southern Thailand. Journal of Maternal-Fetal and Neonatal Medicine 22: 1045-1050.

Jordan S, Lim L, Seubsman SA, Bain C, Sleigh A, et al. (2012) Secular changes and predictors of adult height for 86 105 male and female members of the Thai Cohort Study born between 1940 and 1990. Journal of Epidemiology & Community Health 66: 75-80.

Julia M, van Weissenbruch MM, Delemarre-van de Waal HA and Surjono A (2006) The influence of socioeconomic status on blood pressure of Indonesian prepubertal children. Journal of Human Hypertension 20: 546-548.

Kelles A and Adair L (2009) Offspring consume a more obesogenic diet than mothers in response to changing socioeconomic status and urbanization in Cebu, Philippines. International Journal of Behavioral Nutrition and Physical Activity 6.

Khan NC, Tuyen le D, Ngoc TX, Duong PH and Khoi HH (2007) Reduction in childhood malnutrition in Vietnam from 1990 to 2004. Asia Pacific Journal of Clinical Nutrition 16: 274-278.

Kieu NT, Yasugi E, Hung NT, Kido T, Kondo K, et al. (2002) Serum fatty acids, lipoprotein (a) and apolipoprotein profiles of middle-aged men and women in South Vietnam. Asia Pacific journal of clinical nutrition 11: 112-116.

Kim BK, Choi YS, Oak CH, Park YH, Kim JH, et al. (2012) Determination of thyroid volume by ultrasonography among schoolchildren in Philippines. International Journal of Endocrinology 2012.

King H, Keuky L, Seng S, Khun T, Roglic G, et al. (2005) Diabetes and associated disorders in Cambodia: Two epidemiological surveys. Lancet 366: 1633-1639.

Laillou A, Wieringa F, Tran TN, Van PT, Le BM, et al. (2013) Hypovitaminosis D and mild hypocalcaemia are highly prevalent among young Vietnamese children and women and related to low dietary intake. PLoS ONE [Electronic Resource] 8.

Lee YY, Muda WAMW and Suzuki M (2012) The burden of malnutrition among school children in Kelantan, Malaysia. FASEB Journal Conference: Experimental Biology 20120421.

Lim TO, Ding LM, Zaki M, Merican I, Kew ST, et al. (2000) Clustering of hypertension, abnormal glucose tolerance, hypercholesterolaemia and obesity in Malaysian adult population. The Medical journal of Malaysia 55: 196-208.

Lim TO, Ding LM, Zaki M, Suleiman AB, Fatimah S, et al. (2000) Distribution of body weight, height and body mass index in a national sample of Malaysian adults. Med J Malaysia 55: 108-128.

Lim TO, Rugayah B and Maimunah AH (2004) Familial aggregation and determinants of post challenge blood glucose in four ethnic populations. The Medical journal of Malaysia 59: 357-371.

Mandeep S, Nerenthran L, Chettiar R, Kunsiong D, Gimbo J, et al. (2012) Evidence for rural predominance of Helicobacter pylori: A community based study in Sabah, East Malaysia. Journal of Gastroenterology and Hepatology 27: 20.

Migasena P, Thurnham DI, Pongpaew P, Hongthong K and Harinasuta C (1974) Anthropometric studies in pre-school children in Northeast Thailand. Journal of Nutritional Science & Vitaminology 20: 127-132.

Moench-Pfanner R, Pee Sd, Bloem MW, Foote D, Kosen S, et al. (2005) Food-for-work programs in Indonesia had a limited effect on anemia. Journal of Nutrition 135: 1423-1429.

Mohamad WB, Mokhtar N, Mafauzy M, Mustaffa BE and Musalmah M (1996) Prevalance of obesity and overweight in northeastern peninsular Malaysia and their relationship with cardiovascular risk factors. Southeast Asian Journal of Tropical Medicine & Public Health 27: 339-342.

Narksawat K, Tonmukayakul U and Boonthum A (2009) Association between nutritional status and dental caries in permanent dentition among primary schoolchildren aged 12-14 years, Thailand. Southeast Asian Journal of Tropical Medicine and Public Health 40: 338-344.

Nazri SM, Imran MK, Ismail IM and Faris AA (2008) Prevalence of overweight and self-reported chronic diseases among residents in Pulau Kundur, Kelantan, Malaysia.

Nguyen HT, Eriksson B, Nguyen LT, Nguyen CT, Petzold M, et al. (2012) Physical growth during the first year of life. A longitudinal study in rural and urban areas of Hanoi, Vietnam. BMC pediatrics 12: 26.

Nguyen HT, Eriksson B, Petzold M, Bondjers G, Tran TK, et al. (2013) Factors associated with physical growth of children during the first two years of life in rural and urban areas of Vietnam. BMC Pediatrics 13.

Nguyen PV, Hong TK, Hoang T, Nguyen DT and Robert AR (2013) High prevalence of overweight among adolescents in Ho Chi Minh City, Vietnam. BMC Public Health 13.

Nguyen QN, Pham ST, Do LD, Nguyen VL, Wall S, et al. (2012) Cardiovascular disease risk factor patterns and their implications for intervention strategies in vietnam. International Journal of Hypertension 2012.

Nogueira A, Marcopito L, Lanas F, Galdames D, Jialiang W, et al. (1994) Socio-economic status and risk factors for cardiovascular disease: A multicentre collaborative study in the International Clinical Epidemiology Network (INCLEN). Journal of Clinical Epidemiology 47: 1401-1409.

Norshahida A and Naleena Devi M (2012) The relationship between mothers' educational level and feeding practices among children in selected kindergartens in Selangor, Malaysia: a cross-sectional study. Asian Journal of Clinical Nutrition 4: 39-52.

Oktavianthi S, Trimarsanto H, Febinia CA, Suastika K, Saraswati MR, et al. (2012) Uncoupling protein 2 gene polymorphisms are associated with obesity. Cardiovascular Diabetology 11.

Osman A, Tan TT, Sakinah O, Khalid BAK, Wu LL, et al. (1996) Blood glucose and glycosylated haemoglobin in Malays and Aborigines in Malaysia. Medical Journal of Malaysia 51: 179-187.

Paciorek CJ, Stevens GA, Finucane MM, Ezzati M, Barquera S, et al. (2013) Children's height and weight in rural and urban populations in low-income and middle-income countries: A systematic analysis of population-representative data. The Lancet Global Health 1: e300-e309.

Padang C, Muirden KD, Schumacher HR, Darmawan J and Nasution AR (2006) Characteristics of chronic gout in Northern Sulawesi, Indonesia. Journal of Rheumatology 33: 1813-1817.

Pangaribuan R, Erhardt JG, Scherbaum V and Biesalski HK (2003) Vitamin A capsule distribution to control vitamin A deficiency in Indonesia: effect of supplementation in pre-school children and compliance with the programme. Public Health Nutrition 6: 209-216.

Pedro Ma RA and Barba CVC (2001) Nutritional issues and status of older persons of the Philippines: The IUNS, cronos and other studies. Journal of Nutrition, Health and Aging 5: 92-96.

Phan Si Q, Charles MA, Nguyen Huy C, Le Huy L, Nguyen Anh T, et al. (1994) Blood glucose distribution and prevalence of diabetes in Hanoi (Vietnam). American Journal of Epidemiology 139: 713-722.

Phimmasone K, Douangpoutha I, Fauveau V and Pholsena P (1996) Nutritional status of children in the Lao PDR. Journal of Tropical Pediatrics 42: 5-11.

Pongchaiyakul C, Limpawattana P, Kotruchin P and Rajatanavin R (2013) Prevalence of sarcopenia and associated factors among Thai population. Journal of Bone & Mineral Metabolism 31: 346-350.

Pongchaiyakul C, Nguyen TV, Kosulwat V, Rojroongwasinkul N, Charoenkiatkul S, et al. (2005) Contribution of lean tissue mass to the urban-rural difference in bone mineral density. Osteoporosis International 16: 1761-1768.

Pongchaiyakul C, Nguyen TV, Kosulwat V, Rojroongwasinkul N, Charoenkiatkul S, et al. (2005) Effect of urbanization on bone mineral density: a Thai epidemiological study. BMC Musculoskeletal Disorders 6.

Prodjosudjadi W, Suhardjono, Suwitra K, Pranawa, Widiana IGR, et al. (2009) Detection and prevention of chronic kidney disease in Indonesia: Initial community screening. Nephrology 14: 669-674.

Purwestri RC, Scherbaum V, Inayati DA, Wirawan NN, Suryantan J, et al. (2012) Supplementary feeding with locally-produced Ready-to-Use Food (RUF) for mildly wasted children on Nias Island, Indonesia: comparison of daily and weekly program outcomes. Asia Pacific journal of clinical nutrition 21: 374-379.

Quyen DT, Irei AV, Sato Y, Ota F, Fujimaki Y, et al. (2004) Nutritional factors, parasite infection and allergy in rural and suburban Vietnamese school children. Journal of Medical Investigation 51: 171-177.

Ramli AS, Daher AM, Noor Khan Nor-Ashikin M, Mat-Nasir N, Keat Ng K, et al. (2013) JIS definition identified more malaysian adults with metabolic syndrome compared to the NCEP-ATP III and IDF criteria. BioMed Research International 2013.

Ricci JA and Becker S (1996) Risk factors for wasting and stunting among children in Metro Cebu, Philippines. American Journal of Clinical Nutrition 63: 966-975.

Robson P, Bolton JM and Dugdale AE (1973) The nutrition of Malaysian aboriginal children. The American journal of clinical nutrition 26: 95-100.

Roemling C and Qaim M (2012) Obesity trends and determinants in Indonesia. Appetite 58: 1005-1013.

Ruzita AT, Osman A, Fatimah A and Khalid BAK (1996) Diabetic control among NIDDM patients in urban and rural areas in Malaysia. Medical Journal of Malaysia 51: 48-51.

Rylander C, Phi DT, Odland JO and Sandanger TM (2009) Perfluorinated compounds in delivering women from south central Vietnam. Journal of Environmental Monitoring 11: 2002-2008.

Salibay CC, Dungca JZ and Claveria FG (2008) Serological survey of Toxoplasma gondii infection among urban (Manila) and suburban (Dasmarinas, Cavite) residents, Philippines. Journal of Protozoology Research 18: 26-33.

Sandjaja, Poh BK, Rojroonwasinkul N, Le Nyugen BK, Budiman B, et al. (2013) Relationship between anthropometric indicators and cognitive performance in Southeast Asian school-aged children. British Journal of Nutrition 110.

Saowakontha S, Pongpaew P, Schelp FP, Rojsathaporn K, Intarakha C, et al. (1992) Pregnancy, nutrition and parasitic infection of rural and urban women in Northeast Thailand. Nutrition Research 12: 929-942.

Sari M, De Pee S, Bloem MW, Sun K, Thorne-Lyman AL, et al. (2010) Higher household expenditure on animal-source and nongrain foods lowers the risk of stunting among children 0-59 months old in Indonesia: Implications of rising food prices. Journal of Nutrition 140: 195S-200S.

Schelp FP and Pongpaew P (1985) Analysis of low birth weight rates and associated factors in a rural and an urban hospital in Thailand. Journal of Tropical Pediatrics 31: 4-8.

Schott WB, Crookston BT, Lundeen EA, Stein AD, Behrman JR, et al. (2013) Periods of child growth up to age 8 years in Ethiopia, India, Peru and Vietnam: key distal household and community factors. Social Science & Medicine 97: 278-287.

Selvarajah S, Haniff J, Kaur G, Hiong TG, Cheong KC, et al. (2013) Clustering of cardiovascular risk factors in a middle-income country: a call for urgency. European Journal of Preventive Cardiology 20: 368-375.

Semba RD, de Pee S, Hess SY, Sun K, Sari M, et al. (2008) Child malnutrition and mortality among families not utilizing adequately iodized salt in Indonesia. American Journal of Clinical Nutrition 87: 438-444.

Semba RD, de Pee S, Sun K, Sari M, Akhter N, et al. (2008) Effect of parental formal education on risk of child stunting in Indonesia and Bangladesh: a cross-sectional study. Lancet 371: 322-328.

Senarak W, Chirawatkul S and Markovic M (2006) Health promotion for middle-aged Isan women, Thailand: a participatory approach. Asian Pacific journal of cancer prevention : APJCP 7: 55-59.

Shuhaimi F and Muniandy ND (2012) The association of maternal employment status on nutritional status among children in selected kindergartens in Selangor, Malaysia. Asian Journal of Clinical Nutrition 4: 53-66.

Souganidis ES, Sun K, de pee S, Kraemer K, Rah JH, et al. (2012) Determinants of anemia clustering among mothers and children in Indonesia. Journal of Tropical Pediatrics 58: 170-177.

Suriyaprom K, Harnroongroj T, Namjuntra P, Chantaranipapong Y and Tungtrongchitr R (2007) Effects of tobacco smoking on alpha-2-macroglobulin and some biochemical parameters in Thai males. Southeast Asian Journal of Tropical Medicine and Public Health 38: 918-926.

Suriyaprom K, Namjuntra P, Thawnasom K, Pimainok Y and Tungtrongchitr R (2010) Association between cigarette smoking and metabolic syndrome in Thais. International Journal of Health Research 3: 205-212.

Suriyaprom K, Tungtrongchitr R, Pongpaew P, Phonrat B, Harnroongroj T, et al. (2005) Homocysteine and vitamin status in healthy Thai smokers. Journal of Nutritional and Environmental Medicine 15: 9-21.

Sy RG, Llanes EJB, Reganit PFM, Castillo-Carandang N, Punzalan FER, et al. (2014) Socio-demographic factors and the prevalence of metabolic syndrome among filipinos from the LIFECARE cohort. Journal of Atherosclerosis and Thrombosis 21: S9-S17.

Tengku Hanidza TI, Tunku Khalkausar F, Yasutake A, Zin SM, Hafizan J, et al. (2010) Hair mercury levels in relation to marine fish consumption among adults in Malaysia. (Special Issue: Current issues in environmental quality management in Malaysia.). Environment Asia: 175-185.

Thirukkanesh S and Zahara AM (2010) Compliance to vitamin and mineral supplementation among pregnant women in urban and rural areas in Malaysia. Pakistan Journal of Nutrition 9: 744-750.

Trang NH, Hong TK and Dibley MJ (2012) Active commuting to school among adolescents in Ho Chi Minh City, Vietnam: change and predictors in a longitudinal study, 2004 to 2009. American Journal of Preventive Medicine 42: 120-128.

Vaktskjold A, Van Tri D, Phi DN and Sandanger T (2010) Infant growth disparity in the Khanh Hoa province in Vietnam: a follow-up study. BMC pediatrics 10: 62.

Vliegen K (2000) Report on a nutrition and household food security project in Viet Nam. Food, Nutrition and Agriculture 27: 40-46.

Wagstaff A (2007) The economic consequences of health shocks: Evidence from Vietnam. Journal of Health Economics 26: 82-100.

Yanagisawa Y, Iwamoto S, Kawabata T, Nakamura Y, Charupoonphol P, et al. (2006) Leptin resistance conferred by a combination of single nucleotide polymorphism and the adoption of a Western lifestyle in urban areas of Thailand. Journal of Nutrition, Health & Aging 10: 176-182.

Yiengprugsawan V, Stephan K, McClure R, Kelly M, Seubsman S, et al. (2012) Risk factors for injury in a national cohort of 87,134 Thai adults. Public Health 126: 33-39.

1. **Reviews or conference abstracts (N=16)**

Nutrition survey October-December 1961: Union of Burma. Dept. Defense, Washington 25, D.C. [not specified]. Nutrition survey October December 25.

Aekplakorn W and Mo-Suwan L (2009) Prevalence of obesity in Thailand. Obesity Reviews 10: 589-592.

Al Mamun A (2013) The impact of dietary diversity on obesity development in Asia Oceania. Obesity Research and Clinical Practice 7: 1-2.

Hayati Adilin MAM, McCullough F, Swift J, Norimah AK and Holdsworth M (2011) The role of psychosocial behavioural determinants and the school environment in preventing childhood obesity in terengganu Malaysia. Obesity Reviews 12: 65.

Lipoeto NI, Putra DP, Sulastri D and Jalal F (2013) Blood pressure and food consumption pattern of urban and rural people in west sumatra, Indonesia. Annals of Nutrition and Metabolism 63: 1761-1762.

Ng SW and Popkin BM (2010) Global trends, dynamics and correlates of obesity: A 48-country analysis of repeated surveys. FASEB Journal Conference: Experimental Biology 20100424.

Noor MI, Yusof SM, Koon PB and Karim NA (2013) Habitual energy intake and obesity trend in Malaysia. Obesity Research and Clinical Practice 7: 4-5.

Parikh P, Sandjaja, Poh BK, Rojroongwasinkul N, Khanh LNB, et al. (2013) Anthropometric indicators are associated with nonverbal iq in south east asian school-aged children. Annals of Nutrition and Metabolism 63: 729.

Sakamoto N, Yamborisut U and Yang L (2011) A comparison on the prevalence of being overweight, obese and underweight among children aged 4-6 years between 1997 and 2008. American Journal of Epidemiology 173: S222.

Sandjaja S (2013) Relationship between stunting to deseases, paternal education, and social economy in Indonesian children. Annals of Nutrition and Metabolism 63: 1102-1103.

Singh RB, Suh IL, Singh VP, Chaithiraphan S, Laothavorn P, et al. (2000) Hypertension and stroke in Asia: prevalence, control and strategies in developing countries for prevention. [Review] [121 refs]. Journal of Human Hypertension 14: 749-763.

Sukirna R (2013) Prevalenc of metabolic and behavior cardiovascular risk factors in indonesian population 2007. Annals of Nutrition and Metabolism 63: 1303.

Talib RA, Sharif R and Wan Nazir WN (2013) Television viewing: Impact on body mass index and food intake among school children in Malaysia. Annals of Nutrition and Metabolism 63: 1043.

Teo K, Li W, Chow C, Vaz M, Rangarajan S, et al. (2010) Impact of societal influences on chronic noncommunicable diseases in low, middle, And high income countries: The Prospective Urban Rural Epidemiology (PURE) study. Circulation 122 (2): e177.

Usfar AA, Lebenthal E, Atmarita, Achadi E, Soekirman, et al. (2010) Obesity as a poverty-related emerging nutrition problems: The case of Indonesia. Obesity Reviews 11: 924-928.

Winichagoon P (2013) Thailand nutrition in transition: situation and challenges of maternal and child nutrition. [Review]. Asia Pacific Journal of Clinical Nutrition 22: 6-15.

1. **Not written in English (N=2)**

Badan Penelitian dan Pengembangan Kesehatan, Departemen Kesehatan Republik Indonesia. Laporan Hasil Riset Kesehatan Dasar (RISKESDAS) Indonesia tahun 2007. 2008. CV Kiat Nusa, Jakarta, Indonesia, 2008.

Le Danh T, Le Bach M, Figuie M, Bricas N, Maire B, et al. (2004) Trends in food consumption and in the nutritional status of urban dwellers in Vietnam, over the last twenty years. (Special issue: Food and urbanization) [French]. Cahiers Agricultures 13: 31-38.

1. **Comparison between urban and industrial area (N=1)**

Zailina H, Junidah R, Josephine Y and Jamal HH (2008) The influence of low blood lead concentrations on the cognitive and physical development of primary school children in Malaysia. Asia-Pacific Journal of Public Health 20: 317-326.

1. **Studies using the same dataset as those already included in the review (N=3)**

Aekplakorn W, Chaiyapong Y, Neal B, Chariyalertsak S, Kunanusont C, et al. (2004) Prevalence and determinants of overweight and obesity in Thai adults: Results of the Second National Health Examination Survey. Journal of the Medical Association of Thailand 87: 685-693.

Aekplakorn W, Inthawong R, Kessomboon P, Sangthong R, Chariyalertsak S, et al. (2014) Prevalence and trends of obesity and association with socioeconomic status in Thai adults: National health examination surveys, 1991-2009. Journal of Obesity 2014.

Seubsman SA, Lim LL, Banwell C, Sripaiboonkit N, Kelly M, et al. (2010) Socioeconomic status, sex, and obesity in a large national cohort of 15-87-year-old open university students in Thailand. Journal of epidemiology / Japan Epidemiological Association 20: 13-20.

1. **Waiting for author’s reply (N=6)**

Adair LS (1992) Postpartum nutritional status of Filipino women. American Journal of Human Biology 4: 635-646.

Hla Soe T, Myitzu Tin O, Kyaw Ko Ko H, Zar Chi W, Khin Htet Z, et al. (2011) Prevalence and correlation of obesity, hypertension and type 2 diabetes mellitus in selected townships of Upper Myanmar. Myanmar Health Sciences Research Journal 23: 178-185.

Hoa PT, Khan NC, van Beusekom C, Gross R, Conde WL, et al. (2005) Milk fortified with iron or iron supplementation to improve nutritional status of pregnant women: An intervention trial from rural Vietnam. Food and Nutrition Bulletin 26: 32-38.

Khin Myat T, Han W, Khin Thet W, Theingi T, Zaw M, et al. (2010) Prevalence of hypertension and its associated risk factors in Pintaya Township, Southern Shan State. Myanmar Health Sciences Research Journal 22: 51-57.

Pongpaew P, Saovakontha S, Schelp FP, Supawan V, Hongtong K, et al. (1978) Serum lipid pattern in urban and rural Thai population. Journal of nutritional science and vitaminology 24: 289-296.

Sutanto AH, Sembiring L and Simatupang J (1976) A field survey on ancylostomiasis in school children. [not specified]. Paediatrica Indonesiana 16: 453-463.

# Table S3: Study characteristics of studies conducted in children (<18) from Malaysia, Thailand and Indonesia

| Author | Year of publication | Country | Year of conduct | Sample size | Urban definition | Comparison | Mean age / age range | % Female |
| --- | --- | --- | --- | --- | --- | --- | --- | --- |
| Anuar Zaini^#^ | 2005 | Malaysia | Not stated | 1,045 | Not clearly stated | Not clearly stated | Mean 9.68  9 to10 | 48.9 |
| Sumarni^#^ | 2006 | Malaysia | Not clearly stated | 699 | Urban according to classification by Department of Statistics, Malaysia | Rural according to classification by Department of Statistics, Malaysia | Mean 11.1  10.6 to 12.2 | 48.5 |
| Zalilah^#^ | 2006 | Malaysia | Not clearly stated | 6,555 | Urban based on secondary school categorization by Ministry of Education | Rural based on secondary school categorization by Ministry of Education | 11 to 15.9 | 48.8 |
| Naidu^#^ | 2013 | Malaysia | 2006 | 144 | Urban according to National Health and Morbidity survey (NHMS III) | Rural according to National Health and Morbidity survey (NHMS III) | 7 to 12 | 49.7 |
| Poh^#^ | 2013 | Malaysia | 2011 | 3,542 | Not clearly stated | Not clearly stated | 0.5 to 12.9 | 50.2 |
| Zainuddin^#^ | 2013 | Malaysia | 2008 | 18,078 | Not clearly stated | Not clearly stated | 8 to 10 | Not clearly stated |
| Firestone^#^ | 2011 | Thailand | 2004 | 4,610 | Urban classification to reflect economic and land use pattern in the province | Communities classified as rice growing, plantation, upland and mixed economy strata | 2 to 10 | 48.7 |
| Sakamoto^#^ | 2001 | Thailand | 1997 | 1,157 | Districts in Saraburi municipality | 13 districts outside Saraburi municipality | Mean 5.8  4 to 6 | Not clearly stated |
| Rojroongwasinkul^#^ | 2013 | Thailand | 2011 | 3.119 | Municipal areas | Non-municipal areas | 0.5 to 12.9 | Not clearly stated |
| Julia^#^ | 2004 | Indonesia | 1999 | 2,570 | City of Yogyakarta. Urban subclassified into 2 groups: urban poor (from urban slum) and urban | City of Kidul, about 20 to 40 kms from Yogyakarta | Boys age 6-8.9  Girls age 6-7.9 | 42.3 |
| Sandjaja^#^ | 2013 | Indonesia | 2011 | 7,211 | Not clearly stated | Not clearly stated | 0.5 to 12 | 48.5 |

^#^ studies included in the meta analysis for children

# Table S4: Study characteristics of studies conducted in children (<18) from Laos and Vietnam

| Author | Year of publication | Country | Year of conduct | Sample size | Urban definition | Comparison | Mean age / age range | % Female |
| --- | --- | --- | --- | --- | --- | --- | --- | --- |
| Jurgensen^#^ | 2009 | Laos | 2006 | 621 | Schools in urban area of Vientiane | Schools in semi urban area of Vientiane | 10 to 13 | 52.8 |
| Tuyet | 2003 | Vietnam | 1999 | 348 | First district of Ho Chi Minh City (trading area and majority of people are merchants) | Binh Chanh District (most of people are farmers and fishermen) | 7 to 9 | 100 |
| Leirop^#^ | 2008 | Vietnam | 2004-2005 | 2,546 | Six communities based on socioeconomic characteristics and ecological conditions in Binh Thuan Province | Ten communities based on socioeconomic characteristics and ecological conditions in Binh Thuan Province | Mean 7.5  6 to 10 | Not clearly reported |
| Dang^#^ | 2010 | Vietnam | 1992 | 5,460 | Urban according to General statistical office in both surveys. Status base on the classification at time of each survey | Rural according to General statistical office in both surveys. Status base on the classification at time of each survey | 6 to 15 | 49.3  in 1992 |
|  |  |  | 2000 | 9,870 |  |  |  | 48.7  in 2000 |
| Tang^#^ | 2007 | Vietnam | 2002 | 1,504 | Schools in wealthy urban distracts and less wealth urban district | Schools in semi rural and rural districts | Mean 13.1  11 to 16 | 49.9 |
| Tuan** | 2008 | Vietnam | 1992 | 24,068 | Urban according to General statistical office in both surveys. Status base on the classification at time of each survey | Rural according to General statistical office in both surveys. Status base on the classification at time of each survey | 2 to 65 with separate analysis for 2 to 18 and over 18 | 51.8  in 1992 |
|  |  |  | 2002 | 158,019 |  |  |  | 51.5  in 2002 |
| Le Nguyen^#^ | 2013 | Vietnam | 2011 | 2,872 | Not clearly stated | Not clearly stated | 0.5 to 11 | 49.8 |

^#^ studies included in the meta analysis for children ** the only study conducted in both children and adults

# Table S5: Study characteristics of studies conducted in Adults from Malaysia and Philippines

| Author | Year of publication | Country | Year of conduct | Sample size | Urban definition | Comparison | Mean age / age range | % Female |
| --- | --- | --- | --- | --- | --- | --- | --- | --- |
| Rampal^#^ | 2007 | Malaysia | 2004 | 16,127 | Urban according to Statistics Department of Malaysia | Rural according to Statistics Department of Malaysia | Mean 36.7  15 to 93 | 57.6 |
| Amzi | 2009 | Malaysia | 2002-2003 | 6,766 | Urban according to Malaysian Adult Nutrition Survey (MANS) | Rural according to Malaysian Adult Nutrition Survey (MANS) | 18 to 59 | 50.8 |
| Jinam | 2008 | Malaysia | Not stated | 266 | Temuan and Bidayud communities | Kensiu and Jehai communities | 20 to >70 | 60.1 |
| Suzana^#^ | 2012 | Malaysia | 2006 | 4,676 | Urban according to National Health and Morbidity survey (NHMS III) | Rural according to National Health and Morbidity survey (NHMS III) | 60 to 80+ | 53.4 |
| Mohamud | 2012 | Malaysia | 2006 | 4,341 | Urban according to National Health and Morbidity survey (NHMS III) | Rural according to National Health and Morbidity survey (NHMS III) | 47.8  (SD 14.5) | 64.9 |
| Rasiah | 2013 | Malaysia | 2007 to 2010 | 10,645 | Ten communities from Western Peninsular Malaysia | Nine communities from Eastern Peninsular Malaysia and East Malaysia | 30 and above | Not clearly stated |
| Shariff | 2014 | Malaysia | 2005 to 2009 | 625 | Households from Petaling, Selangor and cities of Kota Bharu and Kuala Lumpur | Households from palm plantations throughout Negeri Sembilan and Kalantan | 19 to 49 | 100 |
| Dahly^#^ | 2010 | Philippines | 2005 | 1,807 | Urbanicity scale. Made up of 7 components: 1. Population size, 2. Population density, 3.Communications, 4 Transportation, 5. Markets, 6 Educational facilities and 7. Health services | | Mean 21 .5  (SD 0.30) | 45.3 |

^#^ Studies included in meta-analysis

# Table S6: Study characteristics of studies conducted in Adults from Thailand

| Author | Year of publication | Country | Year of conduct | Sample size | Urban definition | Comparison | Mean age / age range | % Female |
| --- | --- | --- | --- | --- | --- | --- | --- | --- |
| Aekplakorn^#^ | 2007 | Thailand | 1997 | 3,109 | Urban according to Thai National Health Examination Survey (NHES II and III) | Rural according to Thai National Health Examination Survey (NHES II and III) | 15 to 59 | 64.4 |
|  |  |  | 2004 | 19,133 |  |  |  |  |
| Banwell^#^ | 2009 | Thailand | 2004 | 19,133 | Self report urban location of residence at 10-12 and urban residence in 2005 (U-U) | Self report rural location of residence at 10-12 and in 2005 (R-R) | Median 29  15 to 87 | 52.5 |
| Suriyawong-paisal | 2003 | Thailand | 2000 | 5,305 | Urban according to the Thai Ministry of Interior | Rural according to the Thai Ministry of Interior | Over 35 | 60.5 |
| Jitarin^#^ | 2010 | Thailand | 2004-2005 | 3,163 | Not clearly stated | Not clearly stated | Mean 40.7  (SD 17.2)  18 to 70 | 0 |
| Aekplakorn  (ref 54) | 2011 | Thailand | 2000 | 5,305 | Urban political district | Rural Political district | 50.2 in men  50.6 in women | 60.5 |
| Aekplakorn^#^  (ref 56) | 2011 | Thailand | 2008 | 19,256 | Urban according to Thai National Health Examination Survey (NHES IV) | Rural according to Thai National Health Examination Survey (NHES IV) | 20 to 80+ | 52.5 |

^#^ Studies included in meta-analysis

# Table S7: Study characteristics of studies conducted in adults from Indonesia and Timor-Leste

| Author | Year of publication | Country | Year of conduct | Sample size | Urban definition | Comparison | Mean age / age range | % female |
| --- | --- | --- | --- | --- | --- | --- | --- | --- |
| Koyama | 1988 | Indonesia | 1983 | 212 | Sekeloa, Bandung City | Kampung Tanu, Bandung City | 20 to 50+ | 60.0 |
| Sartika | 2011 | Indonesia | Not clearly stated | 180 | Urban part of City of Depok (25% engaged in agricultural activities) | Rural part of city of Depok (the majority of household engaged in agricultural activity) | Mean 46.4  35.3 to 59.6 | 50.5 |
| Ng^#^ | 2006 | Indonesia | 2001 | 2,927 | Urban according to Purwejo Demographic Surveillance System | Rural area sub classified into quintiles according to an asset survey in 1999 | 15 to 74 | 49.7 |
| Fuke | 2007 | Indonesia | Not clearly stated | 177 | Sangsit | Pedawan | 20 to 60 | 100 |
| Ramke^#^ | 2012 | Timor Leste | 2009-2010 | 2,003 | Urban based on national census data | Rural based on national census data | ≥ 40 | 48.1 |

^#^ Studies included in meta-analysis

# Table S8: Study characteristics of studies conducted in adults from Laos, Vietnam and Myanmar

| Author | Year of publication | Country | Year of conduct | Sample size | Urban definition | Comparison | Mean age / age range | % Female |
| --- | --- | --- | --- | --- | --- | --- | --- | --- |
| Nambooze | 2014 | Laos | 2012 | 144 | Vatluong village | Somsouk and Phouhome village | Over 65 | 61.8 |
| Nguyen^#^ | 2007 | Vietnam | 1992-1993 | 11,981 | All three survey use urban definition according to national census | All three survey use rural definition according to national census | 15 to 51+ | 54.7 |
|  |  |  | 1997-1998 | 15,971 |  |  |  | 54.3 |
|  |  |  | 2001-2002 | 94,656 |  |  |  | 53.2 |
| Hanh | 2001 | Vietnam | 1999 | 300 | Urban area was Ben Thanh ward (district 1) in Ho Chi Minh City | Sub urban area was Phuthuan village (Nha be District) and rural area was Tam Thon Hiep (Can Gio District) in Ho Chi Minh City | 40 to 59 | 62.3 |
| Hanh^#^ | 2001 | Vietnam | 2000 | 217 | Nguyen Cu Trinh Ward,  District 1 | Tan Thanh Dong Village,  Cu Chi District | 60 to 69 | 69.2 |
| Ly | 2013 | Vietnam | 2010 | 1,621 | Urban as determine by government official-the heads of each local commune Health Clinic | Rural and mixed urban/rural communes were defined as those that contain rural areas covering 30% to 50% of their geographic boundary | Mean 52  (SD12.5)  35 to 93 | 56.1 |
| Ha^#^ | 2011 | Vietnam | 2000 | 14,542 | Urban using National Population and Housing census in 1999 | Rural using National Population and Housing census in 1999 | 25 to 64 | 51.2 |
|  |  |  | 2005 | 17,213 |  |  |  | 50.7 |
| Thu Hien | 2013 | Vietnam | 2008 | 1,528 | Not clearly stated | Not clearly stated | Mean 45.6 | Not clearly stated |
| Myo Thet | 1992 | Myanmar | Not stated | 2,611 | Three urban township (Sanchaung, Latha and Pabedan) in Yangon City | Hmawbi Township | Over 15 | 63.4 |

^#^ Studies included in meta-analysis

# Table S9: Results of studies conducted in children from Malaysia

| Author  (year of publication) | Country  (year of conduct) | Obesity definition | Prevalence (%) in urban exposure group (95% CI) | Prevalence (%) in comparison group  (95% CI) | Crude odds ratio (95% CI) | Adjusted odds ratio  (95% CI) | Adjusted factors |
| --- | --- | --- | --- | --- | --- | --- | --- |
| Anuar Zaini^#^  (2005) | Malaysia | BMI >95^th^ percentile for age and sex | 6.5  (5.1 to 8.2) | 5.4  (3.2 to 7.5) | Not clearly reported | 1.22  (0.70 to 2.26) | Age and sex specific criteria for obesity |
| Sumarni^#^  (2006) | Malaysia | Percentiles passing BMI of 25 by International Obesity Task force (IOTF) | 20.8  (17.2 to 24.7) | 23.7  (18.3 to 29.7) | Not clearly reported | 0.85  (0.58 to 1.24) | Age and sex specific criteria for obesity |
|  |  | Percentiles passing BMI of 30 by International Obesity Task force (IOTF) | 7.2  (5.1 to 9.9) | 7.0  (4.1 to 11.1) |  | 1.03  (0.56 to 1.91) |  |
| Zalilah^#^  (2006) | Malaysia | BMI >85^th^ percentile for age and sex base on WHO standard | 19.4  (17.9 to 21.0) | 17.3  (16.1 to 19.5) | Not clearly stated | 1.15  (1.01 to 1.31) | Age and sex specific criteria for obesity |
| Naidu^#^  (2006) | Malaysia | BMI >85^th^ percentile for age and sex base on WHO standard | 22.6  (21.2 to 24.1) | 16.1  (14.7 to 17.5) | 1.53  (1.33 to 1.74) | 1.16  (1.01 to 1.36) | Age and sex specific definition of obesity, ethnicity, guardian BMI status, household income, guardian education |
| Poh^#^  (2013) | Malaysia  (2011) | Z-score based on WHO standard | 12.7 | 8.2 | Not clearly reported | 1.63  (1.29 to 2.06) | Age and sex specific criteria for obesity |
| Zainuddin^#^  (2013) | Malaysia  (2008) | Weight for Age Z-score based on WHO standard | 8.8  (8.0 to 9.8) | 5.9  (5.2 to 6.8) | Not clearly reported | 1.53  (1.10 to 1.77) | Age and sex specific criteria for obesity |
|  |  | BMI for Age Z-score based on WHO standard | 13.0  (11.9 to 14.3) | 8.8  (7.9 to 9.8) |  | 1.55  (1.24 to 1.94) |  |

^#^ Studies included in meta-analysis

# Table S10: Results of studies conducted in children from Thailand and Indonesia

| Author  (year of publication) | Country  (year of conduct) | Obesity definition | Prevalence (%) in urban exposure group (95% CI) | Prevalence (%) in comparison group  (95% CI) | Crude odds ratio (95% CI) | Adjusted odds ratio  (95% CI) | Adjusted factors |
| --- | --- | --- | --- | --- | --- | --- | --- |
| Firestone^#^  (2011) | Thailand (2004) | BMI >95^th^ percentile for age and sex | Not clearly reported | Not clearly reported | 2.66  (1.91 to 3.72) | 1.73  (1.21 to 2.48) | Age and sex specific criteria for obesity, adjustments for household wealth, maternal education, household head occupation, maternal BMI, household size and ethnicity |
| Sakamoto^#^  (2001) | Thailand (1997) | Weight for Height index >97 of the Thai national standard | 22.7  (19.4 to 26.3) | 7.4  (5.4 to 9.9) | Not clearly reported | 3.68  (2.51 to 5.47) | Age and sex specific criteria for obesity |
| Rojroongwasinkul^#^  (2013) | Thailand  (2011) | Z-score based on WHO standard | 11.8 | 5.9 | Not clearly reported | 2.13  (1.62 to 2.79) | Age and sex specific criteria for obesity |
| Julia^#^  (2004) | Indonesia (1999) | Weight for Height Z-score > 2.0 based on WHO standard | 4.1  in non poor urban | 1.0 | Not clearly reported | 4.35  (2.32 to 8.33)  for non poor urban | Age and sex specific criteria for obesity |
|  |  |  | 0.5  in poor urban |  |  | 0.46  (0.51 to 2.09)  for poor urban |  |
|  |  | Percentiles passing BMI of 25 by International Obesity Task force (IOTF) | 4.9  in non-poor urban | 1.0 | Not clearly reported | 5.26  (2.77 to 10.00)  for non poor urban |  |
|  |  |  | 0.7  in poor urban |  |  | 0.69  (0.12 to 2.57)  for poor urban |  |
|  |  | Percentiles passing BMI of 25 by International Obesity Task force (IOTF) | 1.8  in non-poor urban | 0.2 | Not clearly reported | 11.11  (2.56 to 50.0)  for non-poor urban |  |
|  |  |  | 0.0  in poor urban |  |  |  |  |
| Sandjaja^#^  (2013) | Indonesia  (2011) | Z-score based on WHO standard | 5.1 | 1.8 | Not clearly reported | 2.96  (2.21 to 3.99) | Age and sex specific criteria for obesity |

^#^ Studies included in meta-analysis

# Table S11: Results of studies conducted in children from Laos and Vietnam

| Author  (year of publication) | Country  (year of conduct) | Obesity definition | Prevalence (%) in urban exposure group (95% CI) | Prevalence (%) in comparison group  (95% CI) | Crude odds ratio (95% CI) | Adjusted odds ratio  (95% CI) | Adjusted factors |
| --- | --- | --- | --- | --- | --- | --- | --- |
| Jurgensen ^#^  (2009) | Laos  (2006) | Percentiles passing BMI of 25 by International Obesity Task force (IOTF) | 12.0  (8.9 to 16.9) | 5.0  (2.8 to 7.9) | Not clearly reported | 2.65  (1.40 to 5.24) | Age and sex specific criteria for obesity |
| Tuyet  (2003) | Vietnam (1999) | Weight for Height Z-score > 2.0 based on WHO standard | 5.2  (2.2 to 9.9) | 0.0 | Not clearly reported | Not clearly reported |  |
| Leirop^#^  (2008) | Vietnam (2004) | BMI >85^th^ percentile for age and sex base on WHO standard | 4.6  (3.3 to 5.9) | 1.6  (1.0 to 2.2) | Not clearly reported | 2.94  (1.66 to 5.56) | Age and sex specific criteria for obesity |
| Dang^#^  (2010) | Vietnam (1992 and 2000) | Percentiles passing BMI of 25 by International Obesity Task force (IOTF) | 0.7  (0.1 to 1.2)  in 1992 | 0.4 (0.1 to 0.6) in 1992 | Not clearly reported | 1.83 (0.65 to 4.58) in 1992 | Age and sex specific criteria for obesity |
|  |  |  | 6.2  (4.7 to 7.7)  in 2000 | 1.2 (0.9 to 1.5) in 2000 |  | 5.46 (4.09 to 7.28) in 2000 |  |
| Tang^#^  (2007) | Vietnam (2002) | Percentiles passing BMI of 25 by International Obesity Task force (IOTF | 8.2  (4.0 to 12.5)  in wealth urban | 1.6  (0.8 to 2.4)  in semi-rural and rural | Not clearly reported | 5.53  (2.42 to 14.16)  for wealthy urban | Age and sex specific criteria for obesity |
|  |  |  | 5.8  (4.0 to 7.7)  in less wealthy urban |  |  | 3.82  (1.73 to 9.56)  in less wealthy urban |  |
|  |  | Percentiles passing BMI of 30 by International Obesity Task force (IOTF | 0.6  (0.0 to 1.6)  in wealthy urban | 0.2  (0.0 to 0.6)  in semi-rural and rural | Not clearly reported | 2.86  (0.15 to 168.9)  for wealthy urban |  |
|  |  |  | 0.9  (0.2 to 1.7)  in less wealthy urban |  |  | 4.6  (0.56 to 214.3)  in less wealthy urban |  |
| Tuan  (2008) | Vietnam  (1992 and 2002) | BMI >85^th^ percentile for age and sex base on WHO standard age 2-17 | 1.2  (0.5 to 1.9)  in 1992 | 1.4  (0.9 to 1.9)  in 1992 | Not clearly reported | Not clearly reported | Age and sex specific criteria for obesity  Prevalence weighted to be nationally representative |
|  |  |  | 4.7  (4.0 to 5.3)  in 2002 | 1.1  (1.0 to 1.3)  in 2002 |  |  |  |
| Le Nguyen^#^  (2013) | Vietnam  (2011) | Z-score based on WHO standard | 14.3 | 1.4 | Not clearly reported | 11.8  (7.39 to 19.8) | Age and sex specific criteria for obesity |

^#^Studies included in meta-analysis

# Table S12: Results of studies conducted in adults from Malaysia and Philippines

| Author  (year of publication) | Country  (year of conduct) | Obesity definition | | | Prevalence (%) in urban exposure group (95% CI) | Prevalence (%) in comparison group  (95% CI) | Crude odds ratio (95% CI) | Adjusted odds ratio  (95% CI) | Adjusted factors |
| --- | --- | --- | --- | --- | --- | --- | --- | --- | --- |
| Rampal^#^  (2007) | Malaysia (2004) | BMI ≥ 30 | | | 12.0  (11.2 to 12.8) | 11.3  (10.4 to 12.3) | 1.07  (0.94 to 1.21) | 1.16  (1.02 to 1.32) | Age, sex, ethnicity and current smoking |
| Amzi  (2009) | Malaysia (2002) | BMI ≥ 30 | | | 12.0  (10.8 to 13.3) | 12.3  (11.1 to 13.7) | Not clearly reported | Not clearly reported | None |
| Jinam  (2008) | Malaysia | BMI 25-29  in men | | | 25.5 in Bidayuh | 7.1 in Jehai | Not clearly reported | Not clearly reported | Age corrected to 2000 indigenous Malaysian population |
|  |  |  |  |  | 42.2in Temuan | 11.8 in Kensiu |  |  |  |
|  |  | BMI ≥ 30  in men | | | 7.6 in Bidayuh | 0 in Jehai | Not clearly reported | Not clearly reported |  |
|  |  |  |  |  | 18.3 in Temuan | 0 in Kensiu |  |  |  |
|  |  | BMI 25-29  in women | | | 37.7 in Bidayuh | 13.7 in Jehai | Not clearly reported | Not clearly reported | Age corrected to 2000 indigenous Malaysian population |
|  |  |  |  |  | 34.0 in Temuan | 13.8 in Kensiu |  |  |  |
|  |  | BMI ≥ 30  in women | | | 11.0 in Bidayuh | 0 in Jehai | Not clearly reported | Not clearly reported |  |
|  |  |  |  |  | 26.3 in Temuan | 5.1 in Kensiu |  |  |  |
| Suzana^#^  (2012) | Malaysia (2006) | BMI ≥ 25 | | | 44.9  (42.8 to 47.0) | 35.1  (33.0 to 37.2) | Not clearly reported | 1.3  (1.2 to 1.6) | Age restricted population (60-80), adjustments for sex, ethnicity, education, household income and marital status |
|  |  | BMI ≥ 30 | | | 11.5  (10.3 to 12.9) | 9.9  (8.7 to 11.2) | Not clearly reported | 1.1  (0.9 to 1.4) |  |
|  |  | WC ≥ 102 in men  WC ≥ 88 in women | | | 23.6  (21.9 to 25.4) | 18.6  (17.0 to 20.3) | Not clearly reported | 1.2  (1.0 to 1.4) |  |
| Mohamud (2012) | Malaysia (2006) | WC ≥ 90 in men  WC ≥ 80 in women | | | 56.5  (54.4 to 58.6) | 58.4  (56.2 to 60.5) | Not clearly reported | 0.92  (0.82 to 1.04) | Sex specific criteria, not age adjusted |
| Rasiah  (2013) | Malaysia  (2007 to 2010) | BMI ≥ 25 | Highest  Education | University education | 18 | 17 | Not clearly reported | Not clearly reported | Analysis only in men  Age standardized prevalence |
|  |  |  |  | Technical education | 22 | 23 |  |  |  |
|  |  |  |  | Secondary education | 15 | 14 |  |  |  |
|  |  |  |  | Primary education | 14 | 11 |  |  |  |
|  |  |  |  | No education | 9 | 3 |  |  |  |

^#^ Studies included in meta-analysis

**Table S12: Results of studies conducted in adults from Malaysia and Philippines (con.)**

| Author  (year of publication) | Country  (year of conduct) | Obesity definition | | | Prevalence (%) in urban exposure group (95% CI) | Prevalence (%) in comparison group  (95% CI) | Crude odds ratio (95% CI) | Adjusted odds ratio  (95% CI) | Adjusted factors |
| --- | --- | --- | --- | --- | --- | --- | --- | --- | --- |
| Rasiah  (2013) | Malaysia  (2007 to 2010) | BMI ≥ 25 | Highest  Education | University education | 17 | 14 | Not clearly reported | Not clearly reported | Analysis only in women  Age standardize prevalence |
|  |  |  |  | Technical education | 6 | 10 |  |  |  |
|  |  |  |  | Secondary education | 24 | 23 |  |  |  |
|  |  |  |  | Primary education | 24 | 21 |  |  |  |
|  |  |  |  | No education | 20 | 12 |  |  |  |
| Shariff  (2014) | Malaysia  (2005 to 2009) | BMI ≥ 25 | | | Not clearly reported | Not clearly reported | 0.98  (0.72 to 1.35) | Not clearly reported | None |
| Dahly^#^  (2001) ** | Philippines (2005) | BMI ≥ 30 | | | Not clearly reported | Not clearly reported | 1.22  (0.99 to 1.51)  in men | 1.08  (0.85 to 1.32)  in men | Age restricted range, adjustments for assets, income education and marital status |
|  |  |  |  |  |  |  | 1.20  (0.85 to 1.52)  in women | 1.19  (0.93 to 1.51)  in women |  |
|  |  | WC >85 in men | | | Not clearly reported | Not clearly reported | 1.25  (0.99 to 1.57) | 1.06  (0.82 to 1.35) |  |
|  |  | WC >80 in women | | |  |  | 1.27  (0.95 to 1.69) | 1.28  (0.95 to 1.71) |  |

^#^ Studies included in meta-analysis; ** Urban exposure in multivariable regression using urbanicity score (10 points): mean urbanicity score in male 40.6; range 8-61

# Table S13: Results of studies conducted in adults from Thailand

| Author  (year of publication) | Country  (year of conduct) | Obesity definition | Prevalence (%) in urban exposure group (95% CI) | Prevalence (%) in comparison group  (95% CI) | Crude odds ratio (95% CI) | Adjusted odds ratio  (95% CI) | Adjusted factors |
| --- | --- | --- | --- | --- | --- | --- | --- |
| Aekplakorn^#^ (2007) | Thailand  (1997 and 2004) | BMI 25-29  In men | 20.4  17.2 to 24.1  in 1997 | 13.3  (11.4 to 15.5)  in 1997 | Not clearly reported | 1.35  (0.84 to 1.58)  in 1997 | Male only analysis, adjusted for age, geographic region, smoking and marital status |
|  |  |  | 25.1  (23.6 to 26.6)  in 2004 | 16.8  (15.4 to 18.2)  in 2004 |  | 1.56  (1.40 to 1.78)  in 2004 |  |
|  |  | BMI ≥ 30  In men | 7.1  (5.4 to 9.3)  in 1997 | 2.8  (1.7 to 4.7)  in 1997 |  | 1.30  (0.63 to 2.70)  in 1997 |  |
|  |  |  | 7.1  (6.1 to 8.3)  in 2004 | 4.5  (3.7 to 5.6)  in 2004 |  | 1.47  (1.18 to 1.85)  in 2004 |  |
|  |  | WC > 90  in men | 23.4  (18.2 to 29.5)  in 1997 | 10.1  (7.7 to 13.1)  in 1997 |  | 1.35  (0.83 to 2.22)  in 1997 |  |
|  |  |  | 22.7  (21.1 to 24.3)  in 2004 | 13.4  (12.0 to 14.9)  in 2004 |  | 1.58  (1.40 to 1.82)  in 2004 |  |
|  |  | BMI 25-29  in women | 23.9  (22.0 to 26.0)  in 1997 | 22.1  (19.5 to 25.0)  in 1997 | Not clearly reported | 1.36  (1.04 to 1.78)  in 1997 | Female only analysis, adjusted for age, geographic region, smoking and marital status |
|  |  |  | 25.4  (24.1 to 26.7)  in 2004 | 26.9  (25.2 to 28.6)  in 2004 |  | 1.12  (0.99 to 1.26)  in 2004 |  |
|  |  | BMI ≥ 30  in women | 9.9  (8.7 to 11.1)  in 1997 | 7.7  (6.5 to 9.2)  in 1997 |  | 1.31  (0.95 to 1.78)  in 1997 |  |
|  |  |  | 12.3  (11.1 to 13.)  in 2004 | 8.8  (8.0 to 9.8)  in 2004 |  | 1.35  (1.12 to 1.61)  in 2004 |  |
|  |  | WC > 80  in women | 32.0  (29.5 to 34.6)  in 1997 | 29.6  (27.2 to 32.2)  in 1997 |  | 1.35  (1.14 to 1.64)  in 1997 |  |
|  |  |  | 37.2  (34.8 to 39.7)  in 2004 | 36.0  (33.7 to 38.3)  in 2004 |  | 1.10  (0.98 to 1.12)  in 2004 |  |

^#^ Studies included in meta-analysis

**Table S13: Results of studies conducted in adults from Thailand (con.)**

| Author  (year of publication) | Country  (year of conduct) | Obesity definition | Prevalence (%) in urban exposure group (95% CI) | Prevalence (%) in comparison group  (95% CI) | Crude odds ratio (95% CI) | Adjusted odds ratio  (95% CI) | Adjusted factors |
| --- | --- | --- | --- | --- | --- | --- | --- |
| Banwell^#^ (2009) | Thailand (2005) | BMI ≥ 25 | Not clearly reported | Not clearly reported | 1.61 | 1.47  (1.38 to 1.55) | Age, sex, income, education, marital status, ethnicity and region |
| Suriyawongpaisal  (2003) | Thailand  (2000) | BMI ≥ 25 | 43.0  (41.3 to 44.8) | 28.0  (26.1 to 29.9) | 1.94  (1.72 to 2.19) | Not clearly reported | None |
| Jitarin^#^ (2010) | Thailand (2004) | BMI ≥ 23 in men | 38.6  (36.3 to 40.9) | 30.4  (28.0 to 32.9) | 1.43  (1.23 to 1.67) | 1.3  (1.1 to 1.6) | Male only analysis, adjusted for age and marital status |
|  |  | BMI ≥ 23 in women | 44.9  (42.6 to 47.2) | 44.9  (42.3 to 47.5) | Not clearly stated | 1.0  (0.87 to 1.15) | Female only analysis, did not adjust for age |
| Aekplakorn (2011, ref 54) | Thailand (2000) | BMI ≥ 30 | 6.6  (4.4 to 8.8)  in men | 3.1  (1.7 to 4.5)  in men | Not clearly reported | Not clearly reported | Age standardized to Thai population in 2000 |
|  |  |  | 12.6  (10.2 to 14.9)  in women | 9.7  (7.3 to 12.0)  in women |  |  |  |
|  |  | WC > 90 in men | 31.3  (25.4 to 37.2) | 16.2  (9.9 to 22.5) |  |  |  |
|  |  | WC >80 in women | 56.0  (53.1 to 58.9) | 47.5  (40.4 to 54.5) |  |  |  |
| Aekplakorn^#^  (2011, ref 56) | Thailand  (2008) | WC > 90 in men | 28.6  (25.7 to 31.5) | 15.2  (13.6 to 16.8) | Not clearly reported | 2.23  (2.01 to 2.48) | Age standardized to Thai population in 2008, gender specific criteria |
|  |  | WC > 80 in women | 48.1  (47.4 to 50.4) | 43.4  (40.6 to 46.1) | Not clearly reported | 1.21  (1.12 to 1.31) | Age standardized to Thai population in 2008, gender specific criteria |

^#^ Studies included in meta-analysis

# Table S14: Results of studies conducted in adults from Indonesia and Timor-Leste

| Author  (year of publication) | Country  (year of conduct) | Obesity definition | | Prevalence (%) in urban exposure group (95% CI) | Prevalence (%) in comparison group  (95% CI) | Crude odds ratio (95% CI) | Adjusted odds ratio  (95% CI) | Adjusted factors |
| --- | --- | --- | --- | --- | --- | --- | --- | --- |
| Koyama  (1988) | Indonesia  (1983) | BMI ≥ 27 | | 5.5  (1.1 to 15.1)  in men | 1. 0   (0 to 9.0)  in men | Not clearly reported | Not clearly reported | Male only analysis |
|  |  |  |  | 4.4  (1.2 to 11.0)  in women | (1.0)  (0 to 9.5)  in women |  |  | Female only analysis |
| Sartika  (2011) | Indonesia | BMI ≥ 25 | | 57.7  (46.9 to 68.1) | 32.9  (22.7 to 42.9) | 2.88  (1.50 to 5.54) | Not clearly reported | None |
| Ng (2006)^#^ | Indonesia (2000) | BMI ≥ 25 | | 13.3  (9.6 to 18.1)  men  23.7  (19.6 to 28.4)  women | 10.1 (6.2 to 16.1)  men  19.6 (14.5 to 26.1)  women  in richest rural quintile | Not clearly stated | 1.35(0.77 to 2.38)  men  1.13 (0.84 to 1.88)  women | Age and sex |
|  |  |  |  |  | 3.1 (2.2 to 4.2)  men  10.2 (8.3 to 12.5)  women  in middle three quintile |  | 4.35(2.65 to 7.14)  men  2.44 (1.74 to 3.33)  women |  |
|  |  |  |  |  | 0.7 (0.2 to 2.9)  men  2.6 (1.2 to 5.8)  women  in poorest quintile |  | 16.67 (4.35 to 10.0) men  9.09 (4.17 to 20.0) women |  |
| Fuke (2007) | Indonesia  (not stated) | Visceral fat per body weight (cm^2^/kg) as means (SD) | Age 20s | 0.524 (0.186) | 0.576 (0.235) | Not clearly reported | Not clearly reported | Age specific and male only analysis |
|  |  |  | Age 30s | 0.818 (0.278) | 0.617 (0.148) |  |  |  |
|  |  |  | Age 40s | 1.047 (0.299) | 1.098 (0.307) |  |  |  |
|  |  |  | Age 20s | 0.524 (0.186) | 0.576 (0.235) |  |  |  |
| Ramke ^#^  (2012) | Timor-Leste (2009) | BMI ≥ 25 | | Not clearly reported | Not clearly reported | 4.3  (2.9 to 6.3) | 2.9  (1.8 to 4.5) | Age, sex, literacy and household income |
|  |  | BMI ≥ 30 | | Not clearly reported | Not clearly reported | 9.5  (3.5 to 25.8) | 5.0  (1.7 to 15.7) |  |

^#^ Studies included in meta-analysis

# Table S15: Results of studies conducted in adults from Laos, Vietnam and Myanmar

| Author  (year of publication) | Country  (year of conduct) | Obesity definition | Prevalence (%) in urban exposure group (95% CI) | Prevalence (%) in comparison group  (95% CI) | Crude odds ratio (95% CI) | Adjusted odds ratio  (95% CI) | Adjusted factors |
| --- | --- | --- | --- | --- | --- | --- | --- |
| Nambooze  (2014) | Laos  (2012) | BMI ≥ 23 | 16.0 | 3.1 | not clearly reported | 5.78  (1.29 to 35.0) | Age restricted population (over 65) |
| Nguyen^#^  (2007) | Vietnam (1992, 1997 and 2001) | BMI ≥ 25 | 4.8  (4.0 to 5.7)  in 1992 | 1.2  (1.0 to 1.4)  in 1992 | 4.13  (3.18 to 5.39)  in 1992 | 1.79  (1.64 to 1.95)  in 2001 | Age, sex, education, occupation, food expenditure |
|  |  |  | 9.1  (8.3 to 9.9)  in 1997 | 2.3  (2.0 to 2.6)  in 1997 | 4.28  (3.64 to 5.02)  in 1997 |  |  |
|  |  |  | 9.6  (9.3 to 9.9)  in 2001 | 3.5  (3.4 to 3.6)  in 2001 | 2.93  (2.77 to 3.11)  in 2001) |  |  |
| Hanh  (2001) | Vietnam (1999) | BMI ≥ 25 | 17.8  (10.9 to 26.7) | 13.0  (7.1 to 21.2)  in suburban | 1.45  (0.63 to 3.43)  compared to suburban | Not clearly reported | None |
|  |  |  |  | 6.1  (2.2 to 12.7)  in rural | 3.36  (1.20 to 10.78)  compared to rural |  |  |
| Hanh^#^  (2001) | Vietnam  (200) | BMI ≥ 25 | 34.2 in men | 5.6 in men | Not clearly reported | 8.71  (2.73 to 36.0) | Age restricted population (60 to 69) and stratified by sex |
|  |  |  | 25.0 in women | 5.4 in women |  | 5.83  (1.01 to 59.6) |  |
| Ly  (2013) | Vietnam (2010) | BMI ≥ 23 | Not clearly reported | Not clearly reported | Not clearly reported | 1.28  (0.99 to 1.66)  compared to mix urban-rural | Age, systolic blood pressure, diabetes (variable selected using backward stepwise approach) |
|  |  |  |  |  |  | 1.92  (1.0 to 3.70) compared to rural |  |
|  |  | BMI ≥ 25 | Not clearly stated | Not clearly stated | Not clearly reported | 1.41  (1.0 to 2.0)  compared to mix urban-rural | Systolic blood pressure, diabetes, self reported heart attack (variable selected using backward stepwise approach) |
|  |  |  |  |  |  | 2.13  (0.57 to 7.69)  compared to rural |  |

^#^ Studies included in meta-analysis

**Table S15: Results of studies conducted in adults from Laos, Vietnam and Myanmar (con.)**

| Author  (year of publication) | Country  (year of conduct) | Obesity definition | Prevalence (%) in urban exposure group (95% CI) | Prevalence (%) in comparison group  (95% CI) | Crude odds ratio (95% CI) | Adjusted odds ratio  (95% CI) | Adjusted factors |
| --- | --- | --- | --- | --- | --- | --- | --- |
| Ha^#^  (2011) | Vietnam (2000 and 2005) | BMI ≥ 25 | Not clearly reported | Not clearly reported | Not clearly reported | 2.39  (1.70 to 3.19)  in 2000 | Age group, sex, education level and food expenditure |
|  |  |  |  |  |  | 2.08  (1.60 to 2.72)  in 2005 |  |
| Tuan  (2008) | Vietnam  (1992 and 2002) | BMI ≥ 25 | 4.5  (3.4 to 5.6)  in 1992 | 1.1  (0.9 to 1.4)  in 1992 | Not clearly reported | Not clearly reported | Prevalence weighted to be nationally representative |
|  |  |  | 10.0  (9.5 to 10.6_  in 2002 | 3.5  (3.3 to 3.7)  in 2002 |  |  |  |
| Thu Hien  (2013) | Vietnam  (2008) | BMI ≥ 23 | 31.8 | 24.4 | 1.44  (1.14 to 1.82) | 1.39  (1.02 to 1.67) | Education and smoking |
| Myo Thet  (1992) | Myanmar | BMI > 25 | 10.7  (9.0 to 12.6) | 5.9  (4.1 to 6.4) | 2.2  (1.6 to 3.0) | Not clearly reported | None |

^#^ Studies included in meta-analysis

# Table S16: PRISMA checklist

| **Section/topic** | **#** | **Checklist item** | **Reported on page #** |
| --- | --- | --- | --- |
| **TITLE** | | |  |
| Title | 1 | Identify the report as a systematic review, meta-analysis, or both. | 1 |
| **ABSTRACT** | | |  |
| Structured summary | 2 | Provide a structured summary including, as applicable: background; objectives; data sources; study eligibility criteria, participants, and interventions; study appraisal and synthesis methods; results; limitations; conclusions and implications of key findings; systematic review registration number. | 2 |
| **INTRODUCTION** | | |  |
| Rationale | 3 | Describe the rationale for the review in the context of what is already known. | 3 |
| Objectives | 4 | Provide an explicit statement of questions being addressed with reference to participants, interventions, comparisons, outcomes, and study design (PICOS). | 3 |
| **METHODS** | | |  |
| Protocol and registration | 5 | Indicate if a review protocol exists, if and where it can be accessed (e.g., Web address), and, if available, provide registration information including registration number. | n/a |
| Eligibility criteria | 6 | Specify study characteristics (e.g., PICOS, length of follow-up) and report characteristics (e.g., years considered, language, publication status) used as criteria for eligibility, giving rationale. | 4 |
| Information sources | 7 | Describe all information sources (e.g., databases with dates of coverage, contact with study authors to identify additional studies) in the search and date last searched. | 3 |
| Search | 8 | Present full electronic search strategy for at least one database, including any limits used, such that it could be repeated. | Supporting document  Table S1 |
| Study selection | 9 | State the process for selecting studies (i.e., screening, eligibility, included in systematic review, and, if applicable, included in the meta-analysis). | 4 |
| Data collection process | 10 | Describe method of data extraction from reports (e.g., piloted forms, independently, in duplicate) and any processes for obtaining and confirming data from investigators. | 4-5 |
| Data items | 11 | List and define all variables for which data were sought (e.g., PICOS, funding sources) and any assumptions and simplifications made. | 5 |
| Risk of bias in individual studies | 12 | Describe methods used for assessing risk of bias of individual studies (including specification of whether this was done at the study or outcome level), and how this information is to be used in any data synthesis. | 6 |
| Summary measures | 13 | State the principal summary measures (e.g., risk ratio, difference in means). | 5 |
| Synthesis of results | 14 | Describe the methods of handling data and combining results of studies, if done, including measures of consistency (e.g., I^2^) for each meta-analysis. | 6-7 |

| **Section/topic** | **#** | **Checklist item** | **Reported on page #** |
| --- | --- | --- | --- |
| Risk of bias across studies | 15 | Specify any assessment of risk of bias that may affect the cumulative evidence (e.g., publication bias, selective reporting within studies). | 7 |
| Additional analyses | 16 | Describe methods of additional analyses (e.g., sensitivity or subgroup analyses, meta-regression), if done, indicating which were pre-specified. | 6 |
| **RESULTS** | | |  |
| Study selection | 17 | Give numbers of studies screened, assessed for eligibility, and included in the review, with reasons for exclusions at each stage, ideally with a flow diagram. | Figure 1 |
| Study characteristics | 18 | For each study, present characteristics for which data were extracted (e.g., study size, PICOS, follow-up period) and provide the citations. | Page 7 and Supporting file Tables S3-S8 |
| Risk of bias within studies | 19 | Present data on risk of bias of each study and, if available, any outcome level assessment (see item 12). | Page 12 and Supporting file Tables S19-20 |
| Results of individual studies | 20 | For all outcomes considered (benefits or harms), present, for each study: (a) simple summary data for each intervention group (b) effect estimates and confidence intervals, ideally with a forest plot. | Figures 2 and 3; supporting document Tables S9-S15 |
| Synthesis of results | 21 | Present results of each meta-analysis done, including confidence intervals and measures of consistency. | Figures 2 and 3 |
| Risk of bias across studies | 22 | Present results of any assessment of risk of bias across studies (see Item 15). | Page 12 and Supporting file Tables S19-20 and Figures S1-S2 |
| Additional analysis | 23 | Give results of additional analyses, if done (e.g., sensitivity or subgroup analyses, meta-regression [see Item 16]). | Page 9  Figure 4  Table 1 |
| **DISCUSSION** | | |  |
| Summary of evidence | 24 | Summarize the main findings including the strength of evidence for each main outcome; consider their relevance to key groups (e.g., healthcare providers, users, and policy makers). | 10-11 |
| Limitations | 25 | Discuss limitations at study and outcome level (e.g., risk of bias), and at review-level (e.g., incomplete retrieval of identified research, reporting bias). | 12 |
| Conclusions | 26 | Provide a general interpretation of the results in the context of other evidence, and implications for future research. | 12-13 |
| **FUNDING** | | |  |
| Funding | 27 | Describe sources of funding for the systematic review and other support (e.g., supply of data); role of funders for the systematic review. | 14 |

# Table S17: Sensitivity Analysis: Results from random effect analysis meta-regression (Results from Table 1) and trim and fill analysis

| **Stratification** | Randon effect meta-regression | | Trim and fill analysis | |
| --- | --- | --- | --- | --- |
|  | OR for living in an urban environemnt | P-value | OR for living in an urban environemnt | P-value |
| **None** | 1.99 (1.64 to 2.41) | <0.001 | 1.51 (1.34 to 1.71) | <0.001 |
| **Country/countries** |  |  |  |  |
| Philippines and  Malaysia | 1.29 (1.14 to 1.45) | 0.001 | 1.29 (1.28 to 1.43) | <0.001 |
| Thailand | 1.66 (1.30 to 2.11) | 0.001 | 1.47 (1.26 to 1.71) | <0.001 |
| Vietnam and Laos | 3.36 (2.14 to 5.27) | <0.001 | 1.95 (1.31 to 2.87) | <0.001 |
| Indonesia and  Timor-Leste | 3.14 (2.22 to 4.46) | 0.001 | 2.74 (2.10 to 3.59) | <0.001 |
| **Per capita GNI^#^**  **(US dollars)** |  |  |  |  |
| <1,500 | 3.42 (2.42 to 4.84) | <0.001 | 2.03(1.46 to 2.83) | <0.001 |
| 1,500-2,500 | 1.62 (1.20 to 2.18) | <0.001 | 1.38 (1.13 to 1.69) | <0.001 |
| > 2,500 | 1.50 (1.23 to 1.82) | 0.01 | 1.50 (1.30 to 1.72) | <0.001 |
| **Year of field work** |  |  |  |  |
| 2004 to 2013 | 1.85 (1.45 to 2.37) | <0.001 | 1.42 (1.26 to 1.73) | <0.001 |
| Up to 2003 | 2.22 (1.60 to 3.09) | <0.001 | 1.52 (1.20 to 1.94) | <0.001 |
| **Sex of study population** |  |  |  |  |
| Men only | 1.76 (1.14 to 2.73) | 0.020 | 1.69 (1.32 to 2.18) | <0.001 |
| Women only | 1.47 (0.89 to 2.43) | 0.106 | 1.21 (0.95 to 1.56) | <0.001 |
| Both | 2.19 (1.70 to 2.81) | <0.001 | 1.53 (1.30 to 1.80) | <0.001 |
| **Age of population** |  |  |  |  |
| Children | 2.43 (1.72 to 3.43) | <0.001 | 1.52 (1.13 to 2.04) | <0.001 |
| Adults | 1.65 (1.36 to 1.99) | <0.001 | 1.50 (1.33 to 1.79) | <0.001 |
| **Obesity classifcation** |  |  |  |  |
| Non BMIclassifciation  (using WC) | 2.10 (0.53 to 8.28) | 0.145 | 1.21 (0.71 to 2.06) | <0.001 |
| Obesity defined  BMI ≥ 23 or 25 | 2.13 (1.69 to 2.67) | <0.001 | 1.53 (1.33 to 1.78) | <0.001 |
| Obesity defined  as BMI ≥ 30 | 1.39 (0.90 to 2.16) | 0.104 | 1.38 (1.07 to 1.88) | <.0.001 |

Reference groups is living in a rural environment; ^#^GNI gross national income; WC waist circumference; * p-value for heterogeneity chi-square;** Likelihood ratio test for heterogeneity between subgroup by meta-regression, providing F-ratio and p-values

# Table S18: Inter-rater agreement from abstract screening

| Inter-rater agreement | | Reviewer 2 | | |
| --- | --- | --- | --- | --- |
|  |  | Relevant/  Potentially relevant | Not relevant | Total |
| Reviewer 1 | Relevant/  Potentially relevant | 112 | 8 | 120 |
|  | Not relevant | 23 | 558 | 581 |
|  | Total | 135 | 566 | 701 |

588 articles were excluded from abstract reviews and 143 full text articles were assessed for eligibility.

Kappa = 0.85 (Results are shown for articles published up to April 2013)

# Table S19: Summary of bias within studies among children

| Study | Selection bias | Information bias in exposure measurement | Information bias in outcome measurement (BMI) | confounding |
| --- | --- | --- | --- | --- |
| Anuar Zaini^#^ | low risk | unclear risk,  non differential | low risk | low risk |
| Sumarni^#^ | low risk | low risk | low risk | low risk |
| Zalilah^#^ | low risk | low risk | low risk | low risk |
| Naidu^#^ | unclear risk | low risk | low risk | low risk |
| Poh^#^ | low risk | unclear risk | low risk | low risk |
| Zainuddin^#^ | unclear risk | low risk | low risk | low risk |
| Firestone^#^ | low risk | low risk | low risk | low risk |
| Sakamoto^#^ | low risk | low risk | low risk | low risk |
| Rojroongwasinkul^#^ | low risk | low risk | low risk | low risk |
| Julia^#^ | unclear risk | low risk | low risk | low risk |
| Sandjaja^#^ | low risk | unclear risk | low risk | low risk |
| Jurgensen^#^ | low risk | low risk | low risk | low risk |
| Tuyet | low risk | low risk | low risk | low risk |
| Leirop^#^ | low risk | low risk | low risk | low risk |
| Dang^#^ | low risk | low risk | low risk | low risk |
| Tang^#^ | unclear risk | low risk | low risk | low risk |
| Tuan** | unclear risk | low risk | low risk | low risk |
| Le Nguyen^#^ | low risk | unclear risk | low risk | low risk |

^#^ Studies included in meta-analysis; ** Study conducted in both children and adults but reported estimates separately

# Table S20: Summary of bias within studies among adults

| Study | Selection bias | Information bias in exposure measurement | Information bias in outcome measurement (BMI) | confounding |
| --- | --- | --- | --- | --- |
| Rampal^#^ | low risk | low risk | low risk | low risk |
| Azmi | unclear risk | unclear risk, non differential | low risk | unclear risk |
| Jinam | unclear risk | low risk | unclear risk | low risk |
| Suzana^#^ | low risk | low risk | low risk | low risk |
| Mohamud | unclear risk | low risk | low risk | unclear risk |
| Rasiah | unclear risk | low risk | low risk | low risk |
| Shariff | low risk | low risk | low risk | unclear risk |
| Dahly^#^ | low risk | low risk | low risk | low risk |
| Aekplakorn^#^ (ref. 55) | low risk | low risk | low risk | low risk |
| Banwell^#^ | unclear risk | unclear risk, non differential | low risk | low risk |
| Suriyawongpaisal | low risk | low risk | unclear risk | high risk |
| Jitarin ^#^ | unclear risk | unclear risk | unclear risk | low risk |
| Aekplakorn (ref. 54) | low risk | low risk | low risk | low risk |
| Aekplakorn^#^ (ref. 56) | low risk | low risk | low risk | low risk |
| Koyama | unclear risk | low risk | unclear risk | unclear risk |
| Sartika | low risk | low risk | low risk | high risk |
| Ng^#^ | low risk | low risk | low risk | low risk |
| Fuke | unclear risk | low risk | low risk for measurement of visceral fat | low risk |
| Ramke^#^ | low risk | low risk | low risk | low risk |
| Nambooze | low risk | low risk | unclear risk | unclear risk |
| Nguyen^#^ | unclear risk | low risk | unclear risk | low risk |
| Hanh (ref. 42) | unclear risk | low risk | low risk | high risk |
| Hanh^#^ (ref. 45) | unclear risk | low risk | unclear risk | low risk |
| Ly | low risk | low risk | low risk | unclear risk |
| Ha^#^ | low risk | low risk | low risk | low risk |
| Tuan** | unclear risk | low risk | low risk | low risk |
| Thu Hien | low risk | unclear risk | low risk | unclear risk |
| Myo Thet | low risk | low risk | unclear risk | high risk |

^#^ studies included in meta-analysis; ** Study conducted in both children and adults but reported estimates separately
